# Identification of the NRF2 transcriptional network as a therapeutic target for trigeminal neuropathic pain

**DOI:** 10.1126/sciadv.abo5633

**Published:** 2022-08-03

**Authors:** Chirag Vasavda, Risheng Xu, Jason Liew, Ruchita Kothari, Ryan S. Dhindsa, Evan R. Semenza, Bindu D. Paul, Dustin P. Green, Mark F. Sabbagh, Joseph Y. Shin, Wuyang Yang, Adele M. Snowman, Lauren K. Albacarys, Abhay Moghekar, Carlos A. Pardo-Villamizar, Mark Luciano, Judy Huang, Chetan Bettegowda, Shawn G. Kwatra, Xinzhong Dong, Michael Lim, Solomon H. Snyder

**Affiliations:** ^1^ The Solomon H. Snyder Department of Neuroscience, Johns Hopkins University School of Medicine, Baltimore, MD, USA.; ^2^ Department of Neurosurgery, Johns Hopkins University School of Medicine, Baltimore, MD, USA.; ^3^ Department of Molecular and Human Genetics, Baylor College of Medicine, Houston, TX, USA.; ^4^ Jan and Dan Duncan Neurological Research Institute at Texas Children’s Hospital, Houston, TX, USA.; ^5^ Department of Pharmacology and Molecular Sciences, Johns Hopkins University School of Medicine, Baltimore, MD, USA.; ^6^ Department of Psychiatry and Behavioral Sciences, Johns Hopkins University School of Medicine, Baltimore, MD, USA.; ^7^ Department of Neuroscience, Cell Biology, and Anatomy, University of Texas Medical Branch, Galveston, TX, USA.; ^8^ Department of Pathology, Massachusetts General Hospital, Boston, MA, USA.; ^9^ McKusick-Nathans Institute of Genetic Medicine, Johns Hopkins University School of Medicine, Baltimore, MD, USA.; ^10^ Department of Neurology, Johns Hopkins University School of Medicine, Baltimore, MD, USA.; ^11^ Department of Dermatology, Johns Hopkins University School of Medicine, Baltimore, MD, USA.; ^12^ Department of Oncology, Johns Hopkins University School of Medicine, Baltimore, MD, USA.; ^13^ Howard Hughes Medical Institute, Johns Hopkins University School of Medicine, Baltimore, MD, USA.; ^14^ Department of Neurosurgery, Stanford University School of Medicine, Palo Alto, CA, USA.

## Abstract

Trigeminal neuralgia, historically dubbed the “suicide disease,” is an exceedingly painful neurologic condition characterized by sudden episodes of intense facial pain. Unfortunately, the only U.S. Food and Drug Administration (FDA)–approved medication for trigeminal neuralgia carries substantial side effects, with many patients requiring surgery. Here, we identify the NRF2 transcriptional network as a potential therapeutic target. We report that cerebrospinal fluid from patients with trigeminal neuralgia accumulates reactive oxygen species, several of which directly activate the pain-transducing channel TRPA1. Similar to our patient cohort, a mouse model of trigeminal neuropathic pain also exhibits notable oxidative stress. We discover that stimulating the NRF2 antioxidant transcriptional network is as analgesic as inhibiting TRPA1, in part by reversing the underlying oxidative stress. Using a transcriptome-guided drug discovery strategy, we identify two NRF2 network modulators as potential treatments. One of these candidates, exemestane, is already FDA-approved and may thus be a promising alternative treatment for trigeminal neuropathic pain.

## Teaser

Pharmacologically and genetically recruiting the NRF2 transcriptional network alleviates trigeminal neuropathic pain.

## INTRODUCTION

Trigeminal neuralgia is a chronic, debilitating neuropathy characterized by sudden, short, and intense episodes of shooting, stabbing, or shock-like pain in the face (*1*–*3*). The pain can be triggered by activities of everyday life, such as eating, drinking, talking, or brushing teeth. For some patients, even a simple breeze across their face can trigger excruciating pain (*4*, *5*). This pain is so debilitating that trigeminal neuralgia was historically dubbed the “suicide disease” because patients would sometimes take their own life to end their suffering (*6*). Many more bear the pain but endure a poor quality of life, anxiety, and depression (*7*).

Typical trigeminal neuralgia is thought to result from vascular compression of the trigeminal nerve, the principal sensory nerve of the face (*8*, *9*). This compression can injure and demyelinate the nerve, rendering it hyperexcitable and prone to generating ectopic action potentials that may then be interpreted as pain (*10*, *11*). Unfortunately, the current medical treatments for trigeminal neuralgia fall short. The only U.S. Food and Drug Administration (FDA)–approved drug for managing trigeminal neuralgia is the anticonvulsant carbamazepine, which broadly and nonspecifically inhibits neural activity (*12*). Carbamazepine also carries a substantive side effect profile, including hyponatremia and life-threatening drug reactions (*13*–*15*). Patients who fail medical management may resort to surgery, in which microsurgical dissection frees the nerve from the offending artery, or the compressive vein is cauterized and divided (*16*). Microvascular decompression is often effective, with 61 to 80% of patients reporting sustained pain relief years after surgery (*17*). However, this still leaves some patients who experience persistent or recurrent pain. Evidence from long-term follow-up suggests that despite maximum medical and surgical/percutaneous interventions, many patients encounter pain recurrence and incomplete pain control, representing an unmet clinical need (*18*).

To date, there remains an incomplete understanding of the pathophysiologic molecular mechanisms underlying trigeminal neuralgia. Approximately 25% of patients with trigeminal neuralgia do not exhibit vascular compression of the nerve from the outset (*3*, *18*, *19*). About half of these cases may be attributed to secondary causes such as multiple sclerosis or neoplasms, both of which are thought to demyelinate and injure the trigeminal nerve (*20*–*22*). In the other half, the underlying cause remains unknown (*19*, *23*).

These different etiologies appear to ultimately converge upon nerve injury itself. A common consequence of neural injury and inflammation is the generation of reactive oxygen species (ROS), a class of redox-active small molecules (*24*). When uncontrolled, ROS dysregulate cellular processes by inappropriately oxidizing and modifying biomolecules, leading to lipid peroxidation, protein oxidation, and DNA damage (*25*, *26*). Aberrant ROS signaling may contribute to neuropathic pain as well (*27*–*29*). Several ROS, including hydrogen peroxide (H_2_O_2_) and hypochlorite (OCl^−^), can directly activate the pain-transducing channel transient receptor potential ankyrin 1 (TRPA1) (*30*, *31*). In several animal models of sciatic nerve neuropathic pain, antagonizing ROS by administering antioxidants systemically or intrathecally reduces hyperalgesia and relieves allodynia (*27*, *32*). However, the sciatic and trigeminal nerves diverge functionally and transcriptionally in response to painful stimuli, with injury to trigeminal neurons being more intense and more difficult to treat (*33*, *34*).

Here, we report evidence of increased oxidative stress contributing to trigeminal neuropathic pain. A mouse model of trigeminal neuralgia similarly accumulates ROS, several of which directly activate TRPA1. Consistent with previous work describing TRPA1 as a nociceptor in animals (*29*, *35*), we find that pharmacologically inhibiting or genetically eliminating TRPA1 blunts pain. However, attempts at TRPA1 inhibition have been challenging to translate clinically, with multiple failed trials (*36*, *37*). We discover that stimulating the nuclear factor erythroid 2–related factor 2 (NRF2) antioxidant transcriptional network is as powerfully analgesic as directly inhibiting TRPA1 while also reversing underlying oxidative stress. Using a transcriptome-guided drug discovery approach, we identify two NRF2 network modulators as potential treatments. One of these candidates, exemestane, is an FDA-approved drug used to treat estrogen receptor–positive breast cancer and could be readily repurposed as a mechanistically different treatment for trigeminal neuropathic pain.

## RESULTS

### Patients and a mouse model of trigeminal neuralgia exhibit increased oxidative stress

**Fig. 1.**
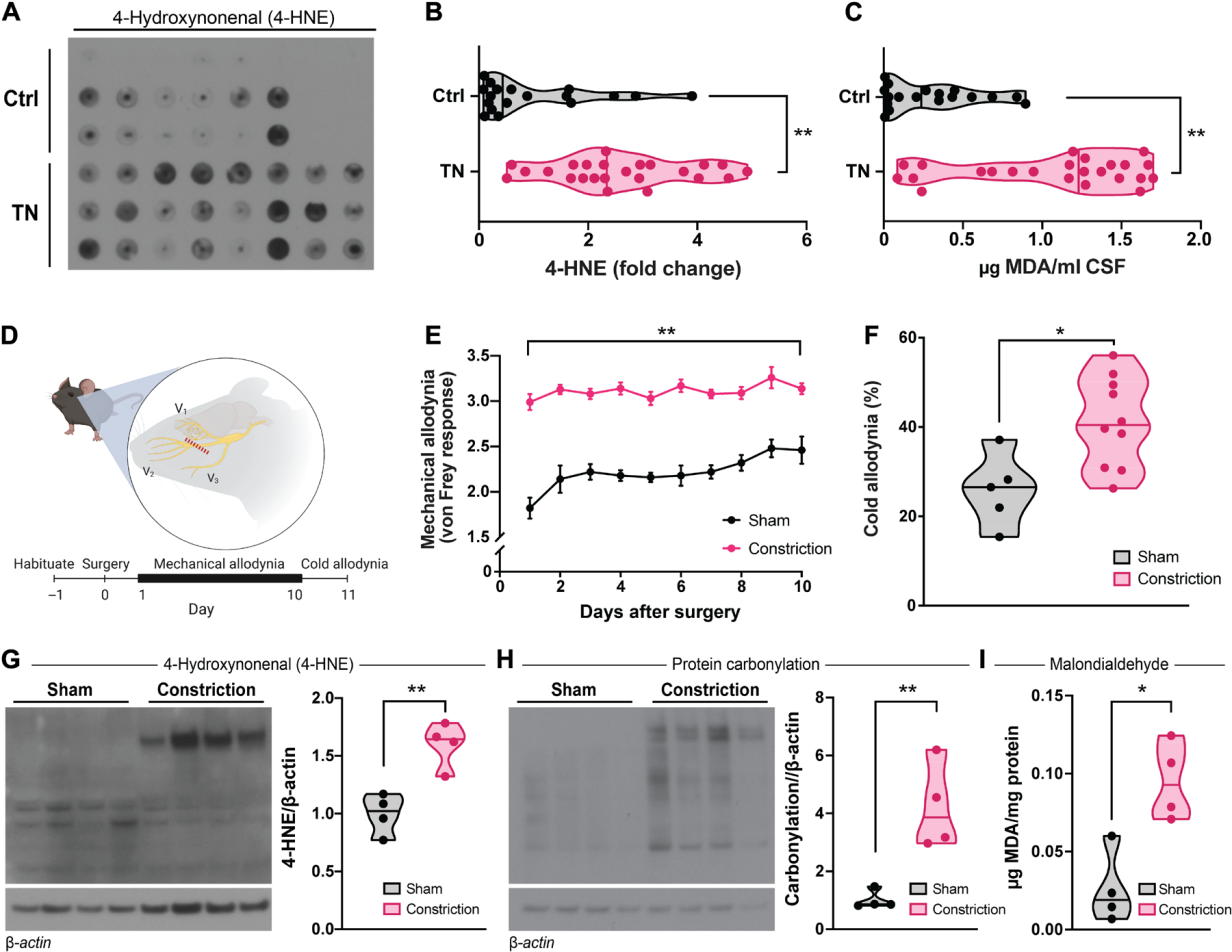
Patients and a mouse model of trigeminal neuralgia exhibit increased oxidative stress. (**A**) Dot blot and (**B**) analysis of relative 4-HNE in CSF from patients with trigeminal neuralgia (TN) normalized to average 4-HNE in CSF from a control population (patients with Chiari malformations, normal pressure hydrocephalus, or pseudotumor cerebri). Points represent individual patients. (**C**) Quantification of MDA in CSF from patients with trigeminal neuralgia and control patients normalized to volume (micrograms of MDA per milliliter of CSF). Points represent individual patients. (**D**) Scheme outlining the constrictive mouse model of trigeminal neuralgia and experimental timeline. One day after habituation, mice underwent constriction of the maxillary nerve or a sham surgery. From the next day onward until day 10, mice were evaluated every day for mechanical allodynia. On day 11, mice were evaluated for cold hypersensitivity. Mice were habituated for 30 min before behavior testing every day. (**E**) Scored mechanical allodynia and (**F**) timed cold allodynia from mice that underwent constriction of the maxillary nerve or sham surgery. Points in (E) represent the means ± SEM of *n* = 5 (sham) and 10 (constriction). Points in (F) represent individual mice. (**G** and **H**) Immunoblots and analysis of (G) 4-HNE and (H) protein carbonylation from maxillary nerves of mice that underwent constriction or sham surgery, normalized to β-actin. Lanes and points represent individual mice. (**I**) Quantification of MDA from maxillary nerves of mice that underwent constriction or sham surgery normalized to protein (micrograms of MDA per milligram of protein). Points represent individual mice. (B, C, and F to I) Median and range depicted. *P < 0.05 and **P < 0.01 by two-tailed unpaired Student’s *t* test.

Microvascular decompression requires a craniotomy, presenting a unique opportunity to sample cerebrospinal fluid (CSF) from patients with trigeminal neuralgia. We evaluated patients’ CSF for evidence of oxidative stress by measuring 4-hydroxynonenal (4-HNE) and malondialdehyde (MDA), two major products of lipid peroxidation (*38*, *39*). Compared to CSF collected from patients undergoing posterior fossa craniectomies (to release Chiari malformations), shunts (to relieve normal pressure hydrocephalus or pseudotumor cerebri), or lumbar punctures (to ease pseudotumor cerebri), both 4-HNE and MDA are elevated in CSF from patients with trigeminal neuralgia (Fig. 1, A to C). CSF 4-HNE and MDA do not correlate with CSF hemoglobin, suggesting that they are not blood contaminants during microvascular decompression (fig. S1, A and B). 4-HNE and MDA are unlikely to be artifacts of surgery or anesthesia either, since both are still elevated in patients with trigeminal neuralgia compared to controls who underwent similar posterior fossa craniectomies (fig. S1, C and D). Instead, the accumulation of CSF 4-HNE and MDA in trigeminal neuralgia may reflect elevated oxidative stress.

To monitor and manipulate the influence of oxidative stress in trigeminal neuralgia, we use a mouse model throughout this study. In this model, branches of the trigeminal nerve are chronically constricted with a loose ligature. As previously reported by several groups (*40*, *41*), constricting branches of the trigeminal nerve in mice elicits allodynia and hyperalgesia in the region innervated by the damaged nerve (Fig. 1D).

Constricting the maxillary division of trigeminal nerve in mice elicits both mechanical and cold allodynia (Fig. 1, E and F). Mice exhibit heightened nocifensive behavior to crude touch with a von Frey filament (Fig. 1E) and to the application of ice-cold acetone to the affected vibrissal pad skin surface (Fig. 1F). Similar to our patient cohort, this mouse model also exhibits notable oxidative stress. Ligating the maxillary nerve results in increased levels of 4-HNE (Fig. 1G), protein carbonylation (Fig. 1H), and MDA (Fig. 1I) in nerve lysates, mirroring the oxidative stress in CSF from patients with trigeminal neuralgia.

### TRPA1 is activated by ROS and mediates trigeminal neuropathic pain

**Fig. 2.**
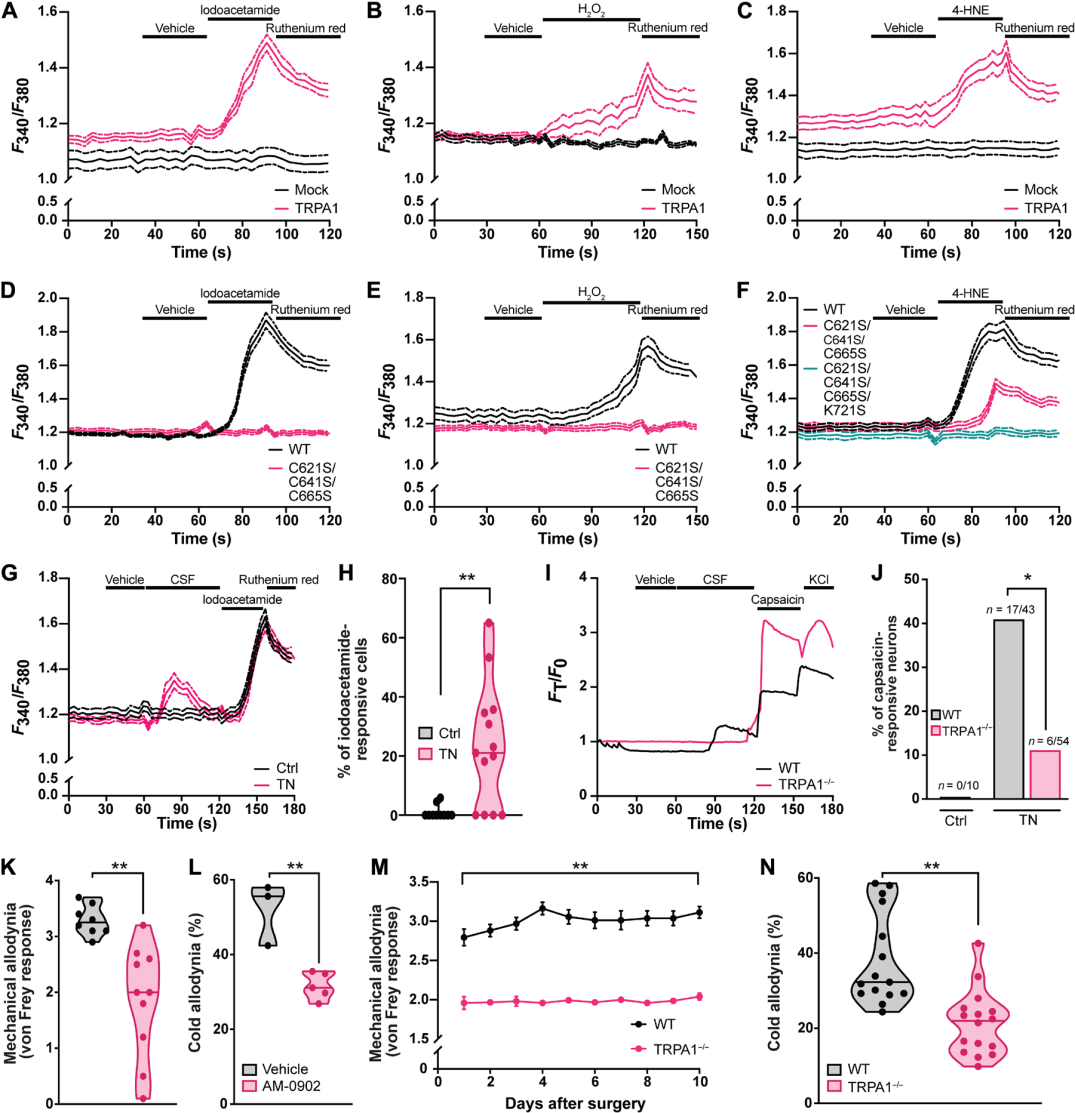
TRPA1 is activated by ROS and mediates trigeminal neuropathic pain. (**A** to **F**) Calcium imaging of HEK-293 cells transiently expressing WT TRPA1 or either (A to C) control vector or (D to F) mutant TRPA1. Either (A and D) 100 μM iodoacetamide, (B and E) 1 mM H_2_O_2_, or (C and F) 100 μM 4-HNE was applied as indicated by black bars. (**G**) Calcium imaging of HEK-293 cells transiently expressing WT TRPA1 in response to CSF from trigeminal neuralgia cases and controls (Ctrl). CSF was diluted into CIB 1:50 before each trial. (**H**) Percent of iodoacetamide-responsive cells activated by CSF from individual control (*n* = 10) or trigeminal neuralgia (*n* = 13) patients. Points represent response to CSF from individual patients. (**I**) Representative traces of Fluo-4 fluorescence from WT and TRPA1^−/−^ trigeminal neurons in response to CSF from patients with trigeminal neuralgia. Pooled CSF was diluted into CIB 1:1 before each trial. (**J**) Percent of WT and TRPA1^−/−^ capsaicin-responsive neurons activated by control/trigeminal neuralgia CSF. (**K**) Scored mechanical allodynia and (**L**) timed cold allodynia from mice following constriction of the maxillary nerve. Mice were treated with either vehicle or AM-0902 (30 mg/kg, p.o.) 30 min before behavior testing. Points in (K) represent the means ± SEM of *n* = 8 (vehicle) and 10 (AM-0902). (**M**) Scored mechanical allodynia and (**N**) timed cold allodynia from WT and TRPA1^−/−^ mice following constriction of the maxillary nerve. Points in (M) represent the means ± SEM of *n* = 16 (WT) and 15 (TRPA1^−/−^). (A to G) Means ± 95% CI depicted with dashed lines. (H, K, and L) Median and range depicted. Points in (L) and (N) represent individual mice. **P < 0.01 by two-tailed unpaired Student’s *t* test. (J) Fraction depicted. *P < 0.05 and **P < 0.01 by Fisher’s exact test.

As ROS accumulate in the constrictive mouse model of trigeminal neuralgia, one mechanism by which they may elicit pain is by activating the pain-transducing channel TRPA1 (*30*, *31*). TRPA1 is a nonselective cation channel located at the plasma membrane of both pain- and itch-encoding sensory neurons (*42*–*44*). TRPA1 not only is a principal sensor of noxious cold (*35*, *42*, *45*, *46*) but also is capable of sensing environmental irritants and reactive molecules such as iodoacetamide (*43*, *47*–*49*). To evaluate whether and how ROS activate TRPA1, we expressed TRPA1 in human embryonic kidney (HEK) 293 cells and monitored changes in intracellular calcium in response to H_2_O_2_ and 4-HNE. Both H_2_O_2_ and 4-HNE activate cells expressing TRPA1 (Fig. 2, A to C), consistent with earlier evidence that TRPA1 is sensitive to redox-active molecules. The nonselective TRP channel inhibitor ruthenium red (*50*) can quench the calcium response, suggesting that H_2_O_2_ and 4-HNE initiate calcium signaling through TRPA1 and not downstream effectors. Neither iodoacetamide, H_2_O_2_, nor 4-HNE elicits a calcium response from cells transfected with the vector backbone alone.

ROS directly activate TRPA1 by covalently bonding or modifying a network of cysteine and lysine residues within the channel. Extensive work by several groups has pinpointed a collection of key residues in TRPA1, including Cys^421^, Cys^621^, Cys^641^, Cys^665^, and Lys^721^ (*49*, *51*, *52*). To test which residues among these sense H_2_O_2_ and 4-HNE, we mutated each individually and in various combinations (fig. S2, A and B). Triply mutating cysteines Cys^621^, Cys^641^, and Cys^665^ to serine renders TRPA1 insensitive to both iodoacetamide and H_2_O_2_ (Fig. 2, D and E) (*51*). As previously described, this triple mutant is still activated by 4-HNE but is no longer sensitive after mutating nearby Lys^721^ (Fig. 2F). These four residues position TRPA1 as both a key sensor and transducer of oxidative stress.

Since TRPA1 senses H_2_O_2_ and 4-HNE in isolation, we wondered whether it may respond similarly to CSF from patients with trigeminal neuralgia. Of the 13 trigeminal neuralgia cases we screened, CSF from all but 4 activates TRPA1-expressing HEK cells. In contrast, CSF from 9 of 11 control patients does not activate TRPA1-expressing cells (*P* = 0.0188) (fig. S3, A to P). Moreover, CSF from patients with trigeminal neuralgia activates cells expressing TRPA1 but not cells transfected with the vector backbone alone (Fig. 2G and fig. S3, I to P), suggesting that components of the CSF specifically activate TRPA1. CSF from each patient with trigeminal neuralgia activates more than 20% of iodoacetamide-responsive cells, whereas CSF from control patients activates less than 2% of iodoacetamide-responsive cells (*P* = 0.0031) (Fig. 2H). To confirm that CSF from patients with trigeminal neuralgia can also activate TRPA1 in its native, neuronal environment, we applied CSF from patients with trigeminal neuralgia and controls to wild-type (WT) and TRPA1-null (TRPA1^−/−^) trigeminal neuronal cultures. To distinguish pain- and itch-encoding neurons from other neurons in both WT and TRPA1^−/−^ cultures, we filtered our analysis to neurons that also responded to the transient receptor potential cation channel subfamily V member 1 (TRPV1) agonist capsaicin (*53*). Previous studies have demonstrated that 97% of TRPA1-positive sensory neurons express TRPV1, and thus, TRPV1 can serve to unbiasedly identify pain- and itch-encoding sensory neurons in the absence of TRPA1 expression (*42*, *48*). By this definition, CSF from patients with trigeminal neuralgia activates almost 40% of WT capsaicin-responsive neurons but only 11% of TRPA1^−/−^ neurons (*P* = 0.0016) (Fig. 2, I and J). CSF from control patients does not activate any sampled WT neurons, further suggesting that unique components of CSF from patients with trigeminal neuralgia can activate endogenous TRPA1.

As endogenous TRPA1 responds to CSF from patients, we tested whether pharmacologically inhibiting or genetically eliminating TRPA1 would blunt pain in the constrictive mouse model of trigeminal neuralgia. We find that WT mice treated with the TRPA1 inhibitor AM-0902 (*54*) exhibit reduced mechanical and cold allodynia (Fig. 2, K and L). Mice that genetically lack TRPA1 exhibit less mechanical and cold allodynia as well, consistent with recent work implicating TRPA1 as a major mediator of trigeminal neuropathic pain in mice (Fig. 2, M and N) (*29*). Both TRPA1^−/−^ males and females exhibit less mechanical and thermal allodynia (fig. S4, A to D).

### Activating NRF2 attenuates trigeminal neuropathic pain and oxidative stress

The potent antinocifensive effects of blocking or eliminating TRPA1 suggest that its inhibition might be a therapeutic strategy in managing trigeminal neuralgia. TRPA1 inhibitors are being actively considered in clinical trials for postoperative pain and diabetic neuropathy (*55*). Unfortunately, several trials have already been abandoned because of disappointing pharmacokinetics or poor efficacy (*36*, *37*). We considered whether we could instead prevent TRPA1 activation by dampening the underlying damaging oxidative stress.

A principal cellular defense mechanism against oxidative or electrophilic stress is activation of the NRF2 antioxidant response (*56*, *57*). NRF2 (or *Nfe2l2*) is a ubiquitously expressed transcription factor that governs the expression of a network of antioxidant genes, including *Nqo1*, *Gsta2*, and *Hmox1* (*58*–*60*). As a master regulator of the cellular redox state, NRF2 is tightly regulated. NRF2 is constitutively expressed but, under basal conditions, is continually tagged for proteasomal degradation by the E3-ubiquitin ligase KEAP1 (*61*, *62*). Similar to TRPA1, KEAP1 harbors several redox-sensitive cysteines that are readily modified by electrophiles and oxidants (*63*–*65*). Oxidation of these cysteines by ROS inhibits KEAP1 (*66*, *67*), stabilizing NRF2 such that it can then translocate to the nucleus to induce the expression of antioxidant and cytoprotective genes.

**Fig. 3.**
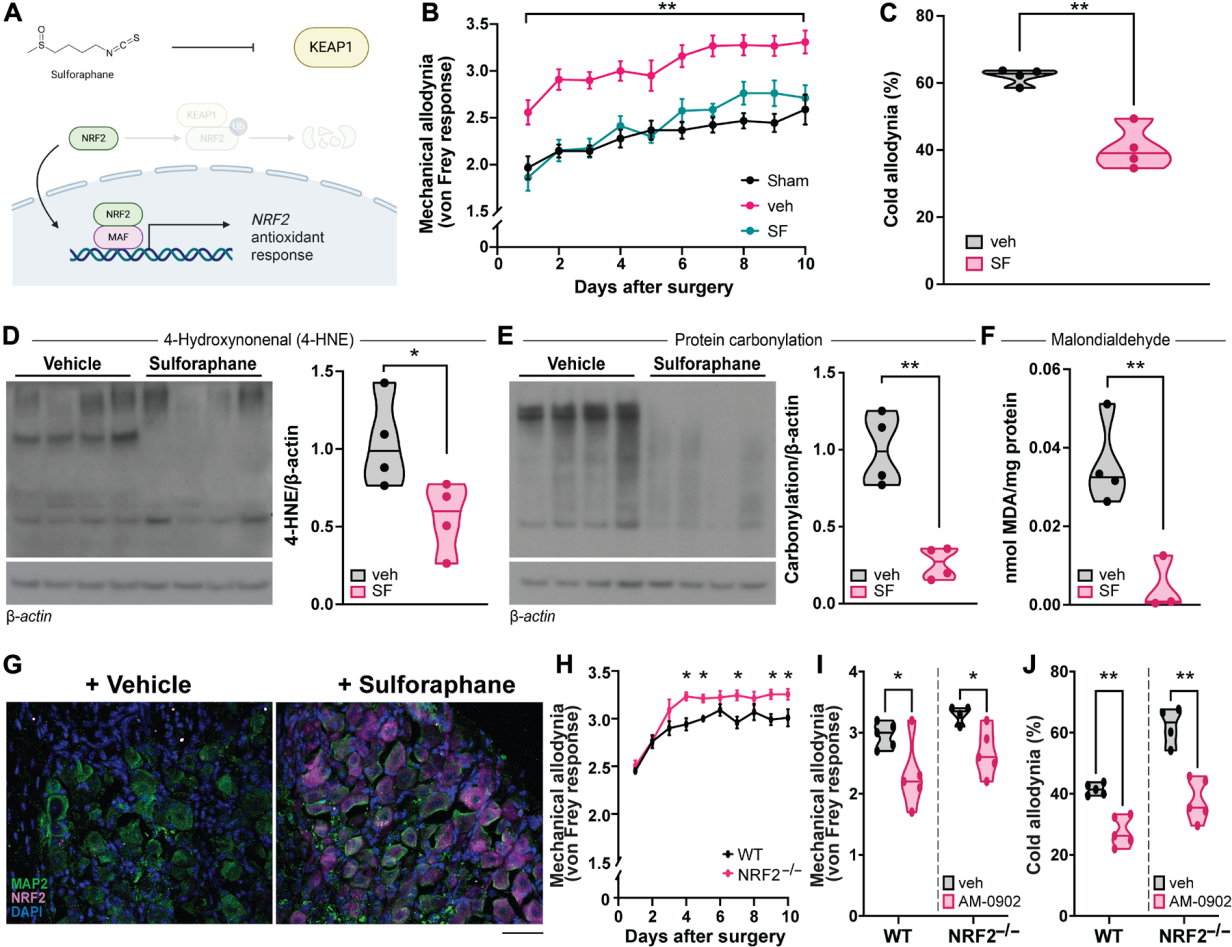
Phamacologically activating NRF2 attenuates trigeminal neuropathic pain and oxidative stress. (**A**) Illustration depicting the mechanism of action of sulforaphane. Sulforaphane inhibits the E3-ubiquitin ligase KEAP1, which normally tags NRF2 for proteasomal degradation. Upon stabilization, NRF2 translocates to the nucleus to induce the expression of antioxidant and cytoprotective genes. (**B**) Scored mechanical allodynia and (**C**) timed cold allodynia from mice that underwent constriction of the maxillary nerve or sham surgery. Mice that underwent constriction were treated with either vehicle (veh) or sulforaphane (SF) (10 mg/kg, i.p.) daily for 2 days before surgery and again daily just after behavior testing. Points in (B) represent the means ± SEM of *n* = 9 (sham), 12 (veh), and 8 (SF). Points in (C) represent individual mice. (**D** and **E**) Immunoblots and analysis of (D) 4-HNE and (E) protein carbonylation from maxillary nerves of mice treated with either veh or SF after nerve constriction, normalized to β-actin. Lanes and points represent individual mice. (**F**) Quantification of MDA from maxillary nerves of mice that underwent constriction or sham surgery normalized to protein (micrograms of MDA per milligram of protein). Points represent individual mice. (**G**) NRF2 immunostaining in trigeminal ganglia from mice treated with vehicle or sulforaphane. MAP2 counterstain identifies neurons, and DAPI identifies nuclei. Scale bar, 50 μm. (**H** and **I**) Scored mechanical allodynia and (**J**) timed cold allodynia from WT and NRF2^−/−^ mice that underwent constriction of the maxillary nerve. Mice in (H) were not treated, whereas mice in (I) and (J) were treated with either veh or AM-0902 (30 mg/kg, p.o.) 30 min before behavior testing. Points in (H) represent the means ± SEM of *n* = 10 (WT) and 10 (NRF2^−/−^). Points in (I) and (J) represent individual mice. (C to F, I, and J) Median and range depicted. *P < 0.05 and **P < 0.01 by two-tailed unpaired Student’s *t* test.

As TRPA1 seems central to pain in the constrictive mouse model of trigeminal neuralgia, we wondered whether activating the NRF2 antioxidant network might lessen allodynia by countering redox stress. Mice treated with the KEAP1 inhibitor sulforaphane (*68*) are less sensitive to both crude touch and cold than vehicle-treated mice after constricting the maxillary nerve (Fig. 3, B and C). However, sulforaphane did not lower mechanical or cold allodynia in TRPA1^−/−^ mice any further compared with genetic deletion of TRPA1 alone, suggesting that oxidative stress contributes to pain upstream of, and possibly through, TRPA1 (fig. S5, A and B). Pretreating mice with sulforaphane limits oxidative stress after nerve constriction and reduces the levels of 4-HNE (Fig. 3D), protein carbonylation (Fig. 3E), and MDA (Fig. 3F). By immunohistochemistry, we find that sulforaphane increases NRF2 expression in neurons of the trigeminal ganglion (Fig. 3G).

Whereas lowering redox stress by activating NRF2 is analgesic, we also considered whether raising redox stress by deleting NRF2 (NRF2^−/−^) can lead to hyperalgesia or allodynia. Consistent with such a model, we found that NRF2^−/−^ mice exhibit greater mechanical and cold allodynia after constriction of the maxillary nerve compared to WT mice (Fig. 3H). Somewhat analogously, Yang *et al.* (*69*) have reported that NRF2^−/−^ mice are hypersensitive to oxaliplatin-induced peripheral neuropathy. Just as with WT mice, however, treating NRF2^−/−^ mice with the TRPA1 antagonist AM-0902 lowers both mechanical and cold allodynia, suggesting that oxidative stress is epistatic to TRPA1, with TRPA1 transducing the increased oxidative stress into hyperalgesia and allodynia (Fig. 3, I and J). NRF2 is also activated by lower concentrations of 4-HNE and H_2_O_2_ than TRPA1, suggesting that TRPA1 is activated during periods of relatively greater oxidative stress. Specifically, we find that 4-HNE stimulates NRF2 at a median effective concentration (EC_50_) of 12 μM [95% confidence interval (CI), 10 to 15 μM] and TRPA1 at 48 μM (95% CI, 32 to 117 μM), whereas H_2_O_2_ triggers NRF2 at an EC_50_ value of 14 μM (95% CI, 11 to 17 μM) and TRPA1 at 133 μM (95% CI, 117 to 167 μM) (fig. S6, A to D).

**Fig. 4.**
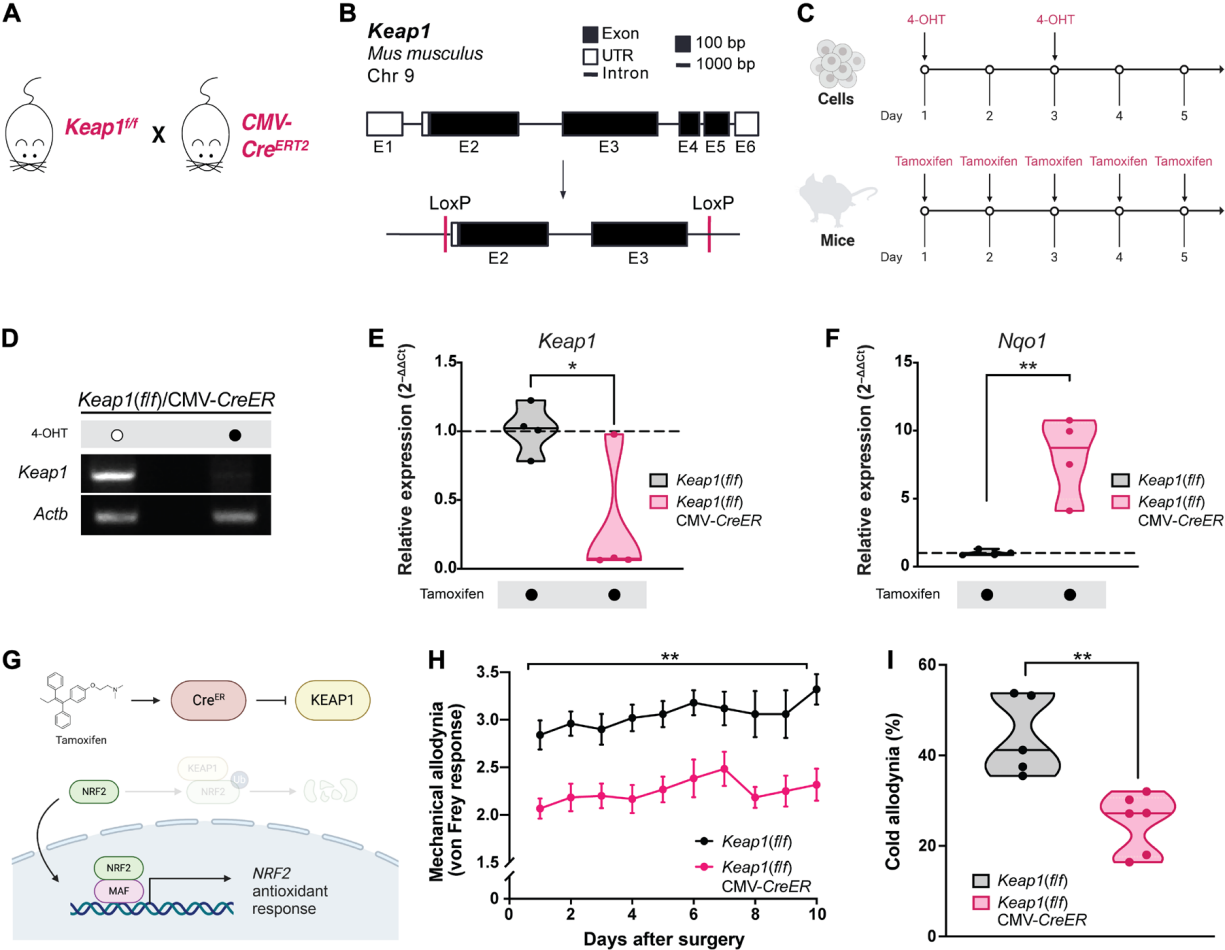
Genetically ablating *Keap1* attenuates trigeminal mechanical and cold allodynia. (**A** and **B**) The *Mus musculus Keap1* gene consists of six exons. To generate a mouse line in which *Keap1* can be deleted, exons 2 and 3 were flanked by two LoxP sites to facilitate their excision by Cre recombinase [Keap1(*f*/*f*)]. To globally and inducibly delete *Keap1*, *Keap1*(*f*/*f*) was crossed to a mouse harboring a tamoxifen-inducible Cre recombinase (CMV-*Cre^ERT2^*) to generate *Keap1*(*f*/*f*)/CMV-*CreER*. (**C**) Top: *Keap1*^*f*/*f*^/CMV-*CreER* fibroblasts were treated with vehicle or 1 μM 4-OHT for 1 day and then again 2 days later. Bottom: *Keap1*^*f*/*f*^/CMV-*CreER* mice were injected intraperitoneally with tamoxifen (75 mg/kg) once every 24 hours over five consecutive days. *Keap1*^*f*/*f*^ (*Cre*-negative) mice were similarly injected with tamoxifen and served as controls. (**D**) PCR of DNA for exons 2 and 3 of *Keap1* from *Keap1*^*f*/*f*^/CMV-*CreER* fibroblasts treated with either vehicle or 4-OHT. (**E** and **F**) Quantitative PCR analysis of (E) *Keap1* and (F) *Nqo1* mRNA normalized to *Actb* from *Keap1*(*f*/*f*) and *Keap1*^*f*/*f*^/CMV-*CreER* mice after treatment with tamoxifen. Points represent individual mice. (**G**) Illustration depicting the mechanism of action of tamoxifen. Tamoxifen permits Cre to translocate to the nucleus, where it excises floxed exons of *Keap1*. Loss of *Keap1* allows Nrf2 to accumulate. Nrf2 then translocates to the nucleus to induce the expression of target genes. (**H**) Scored mechanical allodynia and (**I**) timed cold allodynia from mice that underwent constriction of the maxillary nerve. *Keap1*(*f*/*f*) mice harbor floxed *Keap1* alleles, whereas *Keap1*(*f*/*f*)/CMV-*CreER* mice also harbor a tamoxifen-inducible Cre. Both *Keap1*(*f*/*f*) and *Keap1*(*f*/*f*)/CMV-*CreER* were injected with tamoxifen, and behavioral tests were performed seven or more days after final tamoxifen injection. Points in (H) represent the means ± SEM of *n* = 5 [*Keap1*(*f*/*f*)] and 6 [*Keap1*(*f*/*f*)/CMV-*CreER*]. Points in (I) represent individual mice. (E, F, and I) Median and range depicted. *P < 0.05 and **P < 0.01 by two-tailed unpaired Student’s *t* test.

Although sulforaphane activates NRF2, it can exert additional confounding pharmacologic activities (*70*, *71*). Therefore, we genetically augmented the NRF2 transcriptional network by eliminating KEAP1 altogether. We generated *Keap1*-floxed [*Keap1*(*f*/*f*)] mice that contain a tamoxifen-inducible Cre recombinase (Fig. 4, A and B). Fibroblasts isolated from these mice were treated with vehicle or 4-hydroxytamoxifen (4-OHT) and subsequently genotyped to confirm that *Keap1* is retained unless inducibly targeted and excised (Fig. 4, C and D). When treated with tamoxifen, mice harboring both Cre recombinase and floxed *Keap1* alleles exhibit loss of *Keap1* with a concomitant increase in *Nqo1*, a canonical NRF2 target gene (Fig. 4, E and F). Compared to mice lacking Cre recombinase, mice harboring both Cre and floxed *Keap1* alleles exhibit significantly less mechanical and cold allodynia after nerve constriction (Fig. 4, H and I). Eliminating *Keap1* generally matches the response we observe with sulforaphane, although the analgesic effect of eliminating *Keap1* is slightly more consistent across days than with sulforaphane (Figs. 3B and 4H).

### Drug repositioning identifies NRF2 network modulators as potential treatments for trigeminal neuropathic pain

If limiting oxidative stress can lessen allodynia in patients as it does in mice, then inducing the NRF2 transcriptional network may be an alternative approach to managing trigeminal neuralgia. Unfortunately, sulforaphane exhibits somewhat poor pharmacokinetics that limit its utility in humans (*72*, *73*). To screen for candidate alternatives to sulforaphane, we adapted a transcriptome-guided drug discovery scheme termed transcriptome reversal (*74*, *75*). Transcriptome reversal posits that if a dysregulated transcriptome drives a particular disease, then correcting the transcriptome back toward a normal state may be therapeutic. To reverse the dysregulated transcriptome, the pathologic genetic signature is compared to the transcriptomes of cells treated with different small molecules. Molecules with transcriptome signatures that anticorrelate with the disease signature are prioritized for further validation. The Connectivity Map (CMAP) (*74*, *76*) provides publicly available expression signatures derived from cell lines treated with thousands of small molecules. Transcriptomic approaches that have leveraged CMAP and other resources have successfully identified targeted therapeutics for cancers (*77*), as well as diabetes, inflammatory bowel disease, and neurodevelopmental disorders (*75*, *78*, *79*).

**Fig. 5.**
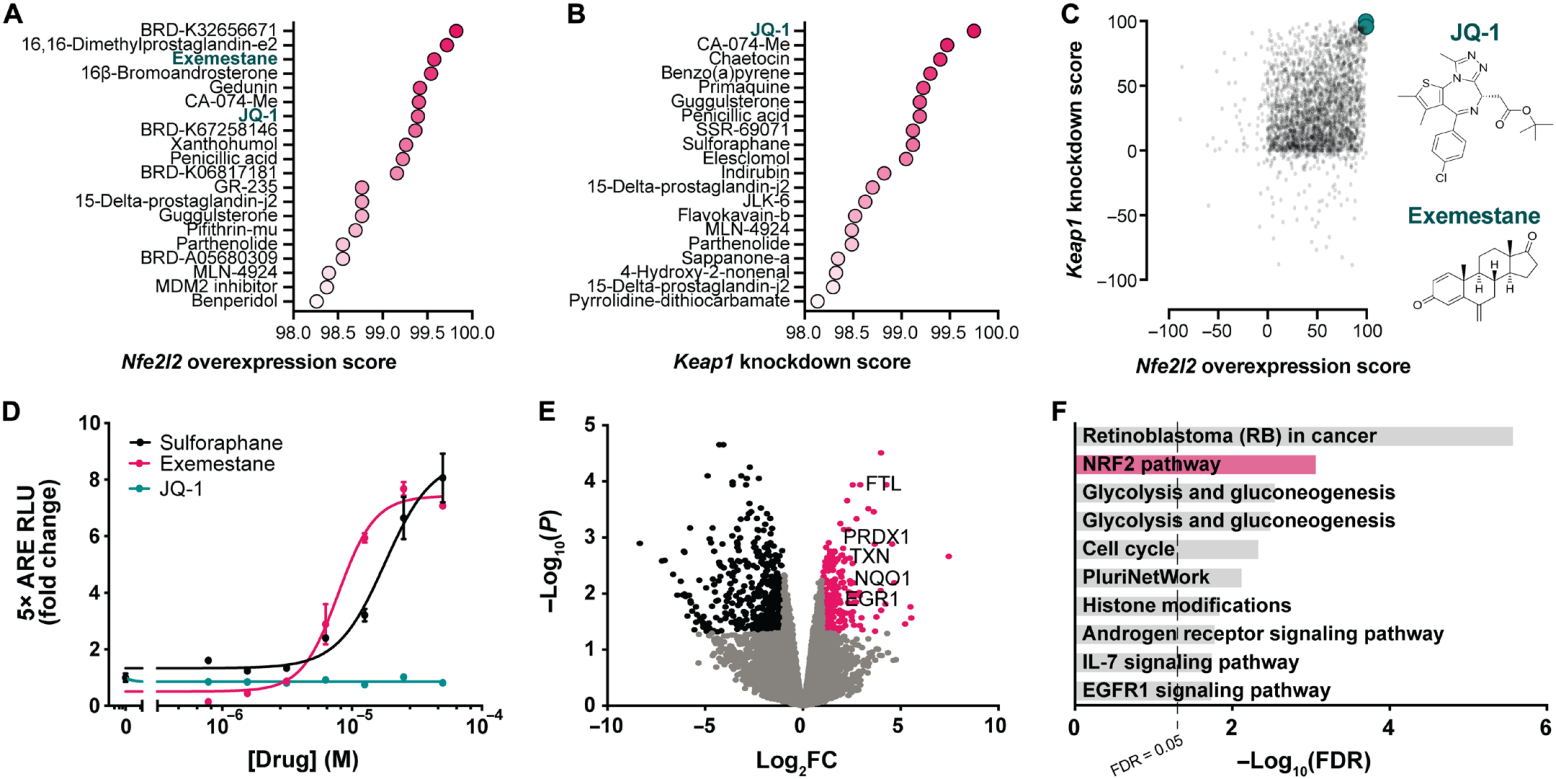
Drug repositioning identifies NRF2 network modulators as potential treatments for trigeminal neuropathic pain. (**A** and **B**) Top 20 compounds with the greatest Connectivity Scores predicted to mimic *Nfe2l2*-derived and *Keap1*-derived transcriptome signatures. (**C**) Plot of Connectivity Scores of molecules in *Nfe2l2*-derived query against the *Keap1*-derived query. Points represent individual molecules. JQ-1 and exemestane are emphasized in green, with their molecular structures to the right. (**D**) Firefly luciferase activity after 6 hours of exposure to varying concentrations of sulforaphane, exemestane, or JQ-1 relative to vehicle treatment, normalized to total protein. Luciferase expression is controlled by a promoter that contains several NRF2 binding sites. *n* = 2 independent experiments in triplicate. (**E**) Volcano plot of transcriptome sequencing of primary human dermal fibroblasts after 48 hours of treatment with vehicle or 0.25 μM JQ-1 (*85*). Points represent individual genes. Black points indicate significantly down-regulated genes [false discovery rate (FDR) < 0.5, log_2_(fold change) ≤ −1], whereas pink points indicate significantly up-regulated genes [FDR < 0.5, log_2_(fold change) ≥ 1]. Gray points are not differentially expressed between treatments. Notable NRF2 target genes are labeled. (**F**) Gene ontology analysis of molecular pathways up-regulated in transcriptome sequencing in (E).

To identify therapeutic candidates that induce the NRF2 transcriptional network, we queried compounds that best mimic the transcriptional signature of overexpressing *Nfe2l2* and genetically silencing *Keap1* (Figs. 3A and 4G and table S1). CMAP scores how well each compound’s transcriptional profile matches the query signature from −100 to +100, with a score of +100 indicating a complete match. In the *Nfe2l2*-queried signature, 187 compounds received a Connectivity Score above CMAP’s recommended cutoff of +90, whereas 114 compounds scored above +90 in the *Keap1*-derived signature (Fig. 5, A and B, and table S1). Among the top 20 candidate compounds prioritized per signature, 7 compounds overlapped between the two queries (Fig. 5C). We focused on exemestane and JQ-1, two overlapping candidate compounds with high scores in both the *Nfe2l2*-derived and *Keap1*-derived signatures. Exemestane is an FDA-approved aromatase inhibitor indicated for the treatment of estrogen receptor–positive breast cancer (*80*, *81*). JQ-1 is an inhibitor of the BET family of bromodomain-containing proteins, displacing them from acetylated lysine residues on histones (*82*). BET inhibitors structurally similar to JQ-1 are being considered in clinical trials to treat a variety of cancers (*83*).

**Fig. 6.**
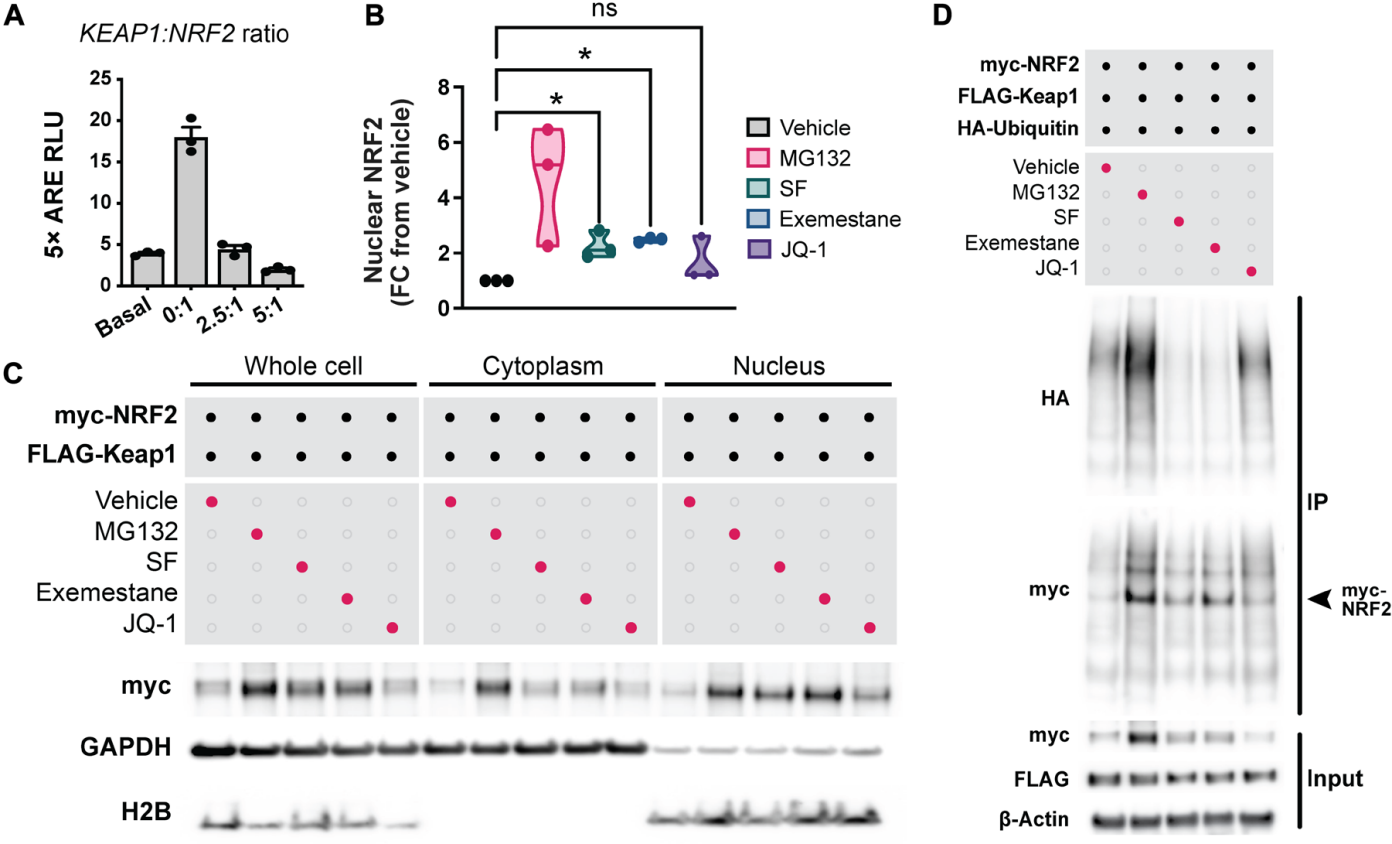
JQ-1’s mechanism of action differs from that of sulforaphane and exemestane. (**A**) To determine a ratio of *KEAP1* to *NRF2* cDNA that best mimics baseline NRF2 activity, luciferase activity was measured 48 hours after transfection with varying ratios of *KEAP1* to *NRF2* cDNA, normalized to total protein. Luciferase expression is controlled by a promoter that contains several NRF2 binding sites and is thus a measure of NRF2 activity. A 2.5 μg of *KEAP1*–to–1 μg of *NRF2* cDNA ratio returned luciferase activity to baseline and was therefore used for subsequent biochemical studies evaluating NRF2 stability and nuclear translocation. (**B** and **C**) Quantification (B) and immunoblots (C) of myc-NRF2, GAPDH (glyceraldehyde-3-phosphate dehydrogenase), and H2B (histone 2B) in whole-cell lysates and cytoplasmic and nuclear subcellular fractions of HEK-293 cells overexpressing FLAG-KEAP1 and myc-NRF2, treated with vehicle, 10 μM MG132 (*N*-carbobenzyloxy-l-leucyl-l-leucyl-l-leucinal), 10 μM sulforaphane (SF), 10 μM exemestane, or 10 μM JQ-1 for 6 hours. Data are expressed as a normalized ratio of nuclear myc-NRF2 compared to the vehicle-treated condition. (**D**) Immunoblots of lysates (input) and myc immunoprecipitate (IP) from HEK-293 cells overexpressing FLAG-KEAP1, myc-NRF2, and HA-ubiquitin. Data are either representative of or quantified from *n* = 3 independent experiments except in (A), where *n* = 1 in triplicate. (A) Means ± SEM depicted. (B) Median and range depicted. *P < 0.05; ns (not significant) indicates *P* > 0.05 by one-way ANOVA followed by post hoc Dunnett’s test.

To test whether exemestane or JQ-1 induces the NRF2 transcriptional network as predicted in silico, we applied both compounds to a reporter cell line in which changes in NRF2 activity are coupled to the expression of firefly luciferase. In these cells, the promoter controlling luciferase expression contains several NRF2 binding sites, thereby directly tying luciferase expression to NRF2 activity. As expected, inhibiting KEAP1 with sulforaphane dose-dependently increases luciferase expression in the NRF2 reporter line. Exemestane similarly increases luciferase expression (Fig. 5D) and up-regulates the canonical NRF2 target *Nqo1* (*84*), suggesting that exemestane also promotes NRF2 transcriptional activity. In contrast, JQ-1 does not stimulate luciferase expression, ostensibly suggesting that it does not induce the NRF2 transcriptional network (Fig. 5D). However, subsequent reanalysis of our published RNA sequencing data (*85*) uncovered that JQ-1 unexpectedly up-regulates a number of canonical NRF2 target genes in primary human dermal fibroblasts, such as *NQO1*, *FTL*, *PRDX1*, *TXN*, and *EGR1* (Fig. 5E and table S2). JQ-1 also up-regulates *Nqo1* and *Hmox1* in mouse corneal fibroblasts and monocytes (*86*, *87*). Unbiased gene ontology and genetic network analyses detected that the NRF2 pathway is the second most up-regulated pathway after treatment with JQ-1 (Fig. 5F and table S3). JQ-1 thus induces much of the NRF2 transcriptional network, but by a mechanism that may not involve classical NRF2 signaling. Consistent with this, we find that JQ-1 neither stabilizes NRF2 nor inhibits its ubiquitination, whereas both sulforaphane and exemestane do. Both sulforaphane and exemestane also promote nuclear translocation of NRF2, but JQ-1 does not (Fig. 6, A to D). JQ-1 might instead up-regulate antioxidant genes by remodeling chromatin through BET proteins, stimulating NRF2 in an unconventional manner (*86*, *88*), or activating alternative transcription factors (*89*), but the exact mechanisms remain presently unclear.

**Fig. 7.**
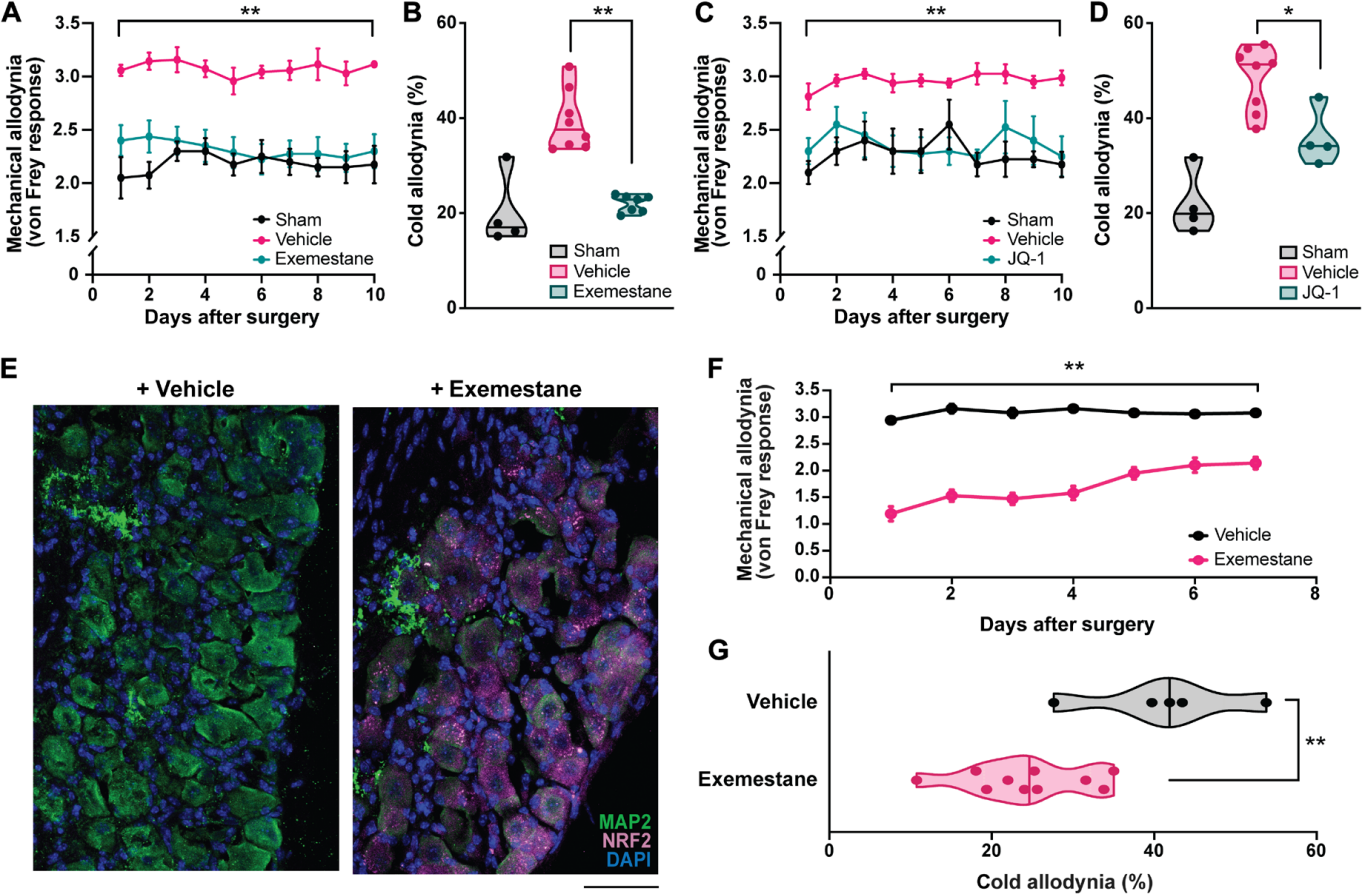
Identified NRF2 network modulators exemestane and JQ-1 alleviate mechanical and cold allodynia. (**A** and **C**) Scored mechanical allodynia and (**B** and **D**) timed cold allodynia from mice that underwent constriction of the maxillary nerve or sham surgery. Mice that underwent constriction were treated with either vehicle, exemestane (10 mg/kg, i.p.), or JQ-1 (40 mg/kg, i.p.) daily for 2 days before surgery and again daily just after behavior testing. Points in (A) represent the means ± SEM of *n* = 4 (sham), 7 (vehicle), and 8 (exemestane). Points in (C) represent the means ± SEM of *n* = 4 (sham), 8 (vehicle), and 4 (JQ-1). Points in (B) and (D) represent individual mice. (**E**) NRF2 immunostaining in trigeminal ganglia from mice treated with vehicle or exemestane. MAP2 counterstain identifies neurons, and DAPI identifies nuclei. Scale bar, 50 μm. (**F**) Scored mechanical allodynia and (**G**) timed cold allodynia from mice that underwent constriction of the maxillary nerve after a single, local treatment of vehicle or exemestane (25 μg per site) to the maxillary nerve. Points in (F) represent the means ± SEM of *n* = 5 (vehicle) and 10 (exemestane). Points in (G) represent individual mice. (B, D, and G) Median and range depicted. *P < 0.05 and **P < 0.01 by two-tailed unpaired Student’s *t* test.

As both exemestane and JQ-1 recruit the NRF2 transcriptome, we evaluated whether they could lower allodynia in the constrictive mouse model of trigeminal neuralgia. Just as with sulforaphane, mice treated with either exemestane or JQ-1 are much less sensitive to both crude touch (Fig. 7, A and C) and the application of ice-cold acetone to the ligated vibrissal pad skin surface (Fig. 7, B and D). By immunohistochemistry, we find that exemestane robustly increases NRF2 expression in neurons of the trigeminal ganglion (Fig. 7E). Directly applying exemestane to the maxillary nerve similarly lessens both mechanical and cold allodynia (Fig. 7, F and G), suggesting that exemestane may also exert analgesia locally at the nerve.

**Fig. 8.**
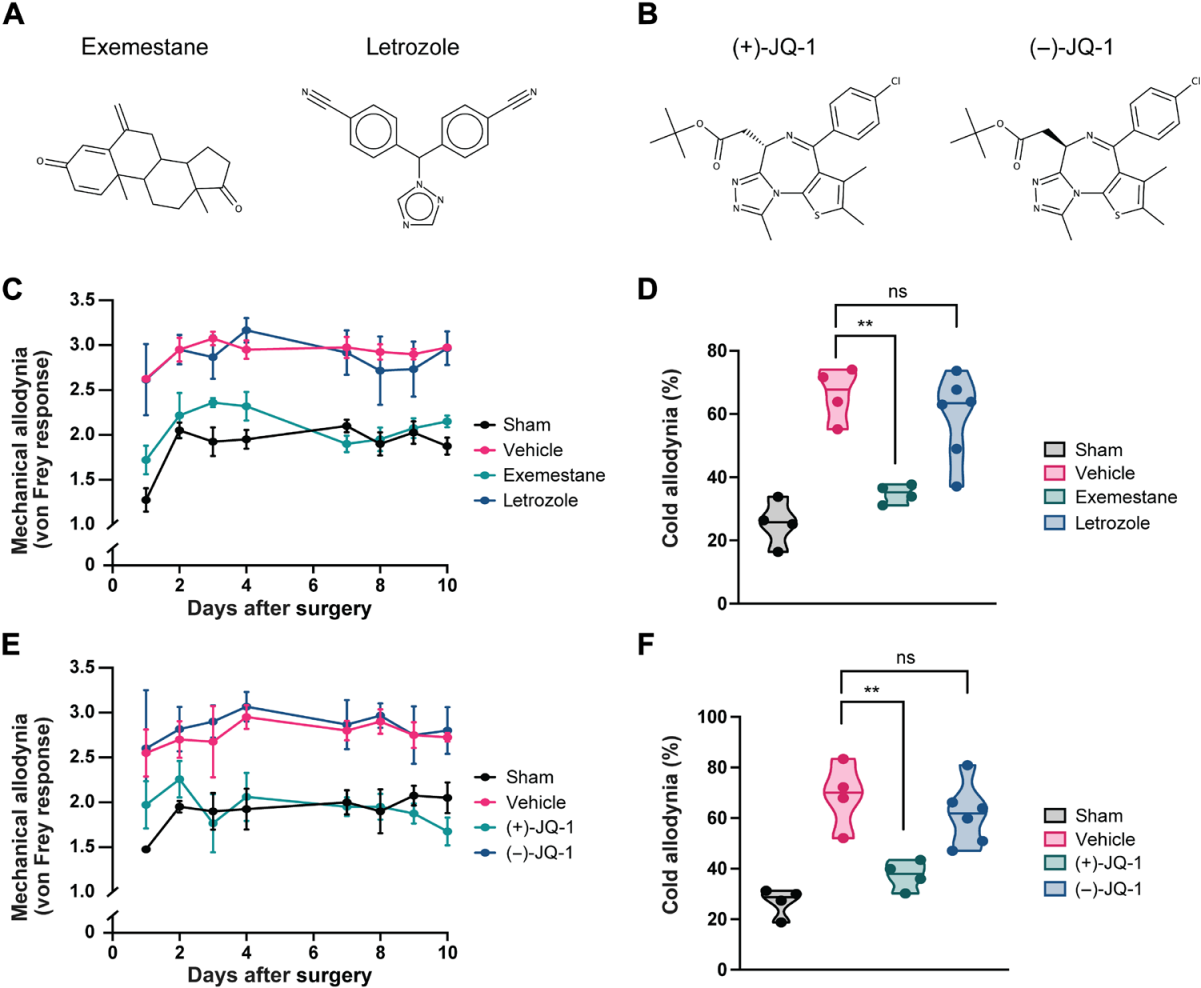
Neither letrozole nor (−)-JQ-1 replicates the analgesic effects of exemestane or (+)-JQ-1. (**A** and **B**) Molecular structures of (A) exemestane and letrozole and (B) (+)-JQ-1 and (−)-JQ-1. (**C** and **E**) Scored mechanical allodynia and (**D** and **F**) timed cold allodynia from mice that underwent constriction of the maxillary nerve or sham surgery. Mice that underwent constriction were treated with either vehicle, exemestane (10 mg/kg, i.p.), letrozole (10 mg/kg, i.p.), (+)-JQ-1 (40 mg/kg, i.p.), or (−)-JQ-1 (40 mg/kg, i.p.) daily for 2 days before surgery and again daily just after behavior testing. Points in (C) represent the means ± SEM of *n* = 4 (sham), 4 (vehicle), 6 (exemestane), and 6 (letrozole). Points in (E) represent the means ± SEM of *n* = 4 (sham), 4 (vehicle), 7 [(+)-JQ-1], and 7 [(−)-JQ-1]. Points in (D) and (F) represent individual mice. (D and F) Median and range depicted. **P < 0.01; ns (not significant) indicates *P* > 0.05 by two-tailed unpaired Student’s *t* test.

To assess whether exemestane or JQ-1 acts through alternative off-target mechanisms, we also evaluated nocifensive behavior in WT mice treated with either letrozole or (−)-JQ-1. Similar to exemestane, letrozole is a potent aromatase inhibitor but is structurally unrelated (*90*) and does not induce *Nqo1* (*84*). (−)-JQ-1 is the inactive stereoisomer of (+)-JQ-1 (Fig. 8, A and B) (*82*). We observe that while exemestane and (+)-JQ-1 lower both mechanical and cold allodynia, neither letrozole nor (−)-JQ-1 does (Fig. 8, C to F). The analgesic activity of exemestane and JQ-1 is thus less likely due to alternative effects from aromatase inhibition or nonspecific changes in DNA topology. To next test whether sulforaphane, exemestane, and JQ-1’s analgesic activities are dependent on NRF2, we treated NRF2^−/−^ mice with all three drugs. We find that both sulforaphane and exemestane lose their analgesic effects in NRF2^−/−^ mice (fig. S7, A to D), suggesting that their mechanisms of action require NRF2. Curiously, JQ-1 still retains some of its analgesic activity. JQ-1 lowers both mechanical and cold allodynia in NRF2^−/−^ mice (fig. S7, E and F), although the effects are more muted in comparison to WT mice. Considering that JQ-1 does not biochemically influence NRF2 in the same manner as sulforaphane and exemestane (Fig. 6), this further suggests that how JQ-1 up-regulates the NRF2 transcriptional network may only partially depend on NRF2 itself. Whether JQ-1 stimulates other transcription factors or chromatin remodeling (*89*) will require future investigation.

Neither sulforaphane, exemestane, nor JQ-1 inhibits TRPA1 channel activity, suggesting that their analgesic effects are not mediated by directly inhibiting the nociceptor itself (fig. S8, A to C). Sulforaphane, exemestane, and JQ-1 do not affect TRPA1 expression either (fig. S9, A to H). Instead, exemestane and JQ-1 may limit oxidative stress. Similar to sulforaphane, pretreating mice with exemestane or JQ-1 reduces the levels of 4-HNE (fig. S10, A and C) and protein carbonylation (fig. S10, B and D) after nerve constriction.

We considered whether bypassing NRF2 and directly administering an antioxidant might also reduce allodynia. Several groups have reported that antioxidants can lower hyperalgesia and allodynia in other animal models of neuropathic pain like sciatic chronic constriction injury (*27*) and spinal nerve constriction (*91*), but the effect is notably acute and only lasts a few hours. In contrast, the effects of sulforaphane, exemestane, and JQ-1 persist 24 hours after treatment. We find that treating mice with the antioxidant ascorbate does not reduce either mechanical or cold allodynia (fig. S11, A and B) or the levels of 4-HNE and protein carbonylation (fig. S11, C and D) at 24 hours, underscoring the value of specifically targeting the NRF2 antioxidant network in sustaining analgesia. Because most small-molecule antioxidants such as ascorbate act by stoichiometrically scavenging ROS directly, they are likely most effective when administered very frequently and at high dosages.

## DISCUSSION

Trigeminal neuralgia is a chronic, debilitatingly painful condition. Unfortunately, medical treatments for trigeminal neuralgia often fall short, in part because the pathophysiology is incompletely understood. Currently, the only FDA-approved drug for managing trigeminal neuralgia is the anticonvulsant carbamazepine, which broadly and nonspecifically inhibits neural activity (*12*, *14*). However, carbamazepine carries a notable side effect profile, including hyponatremia, leukopenia, ataxia, and the risks of drug reaction with eosinophilia and systemic symptoms and Stevens-Johnson syndromes (*13*–*15*). Other drugs with fewer side effects such as gabapentin, pregabalin, and antidepressants are sometimes prescribed off-label but are less effective than carbamazepine (*17*). Up to 20 to 40% of patients with trigeminal neuralgia are also prescribed opiates for their pain despite little evidence supporting their use (*92*, *93*), potentially worsening the socioeconomic and medical burdens of opiate overdose and addiction. Alternative treatments such as radiofrequency and/or glycerin rhizotomy or stereotactic radiosurgery use heat, chemicals, or radiation to ablate the trigeminal nerve to blunt pain. However, these procedures can leave patients with postprocedural numbness or devastating anesthesia dolorosa. In light of these limitations, we sought to better understand the pathophysiology underlying trigeminal neuralgia.

**Fig. 9.**
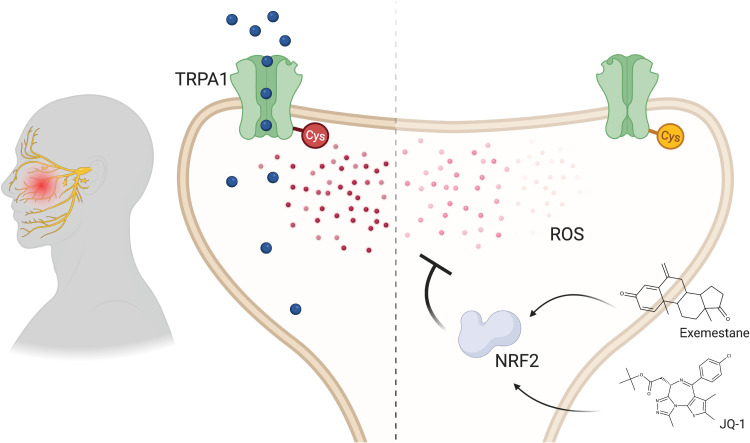
Model by which activation of the NRF2 transcriptional network is analgesic in trigeminal neuropathic pain. Patients and a mouse model of trigeminal neuropathic pain exhibit increased oxidative stress, as evidenced through elevated levels of ROS (red). As ROS accumulate, one mechanism by which they may elicit pain is by activating the pain-transducing channel TRPA1 (green). ROS directly activate TRPA1 by covalently bonding or modifying a network of cysteine and lysine residues within the channel (Cys). The transcription factor NRF2 (gray) is a master regulator of the cellular redox state, governing the expression of a network of antioxidant genes. Recruiting the NRF2 transcriptome pharmacologically with the small molecules exemestane or JQ-1 lowers oxidative stress, thereby alleviating mechanical and cold allodynia in the mouse model of trigeminal neuropathic pain.

By leveraging a combined clinical, molecular, and computational approach, the present study identifies the NRF2 transcriptional network as a potential therapeutic target for trigeminal neuropathic pain (Fig. 9). Using a transcriptome-guided drug discovery approach, we identify exemestane and JQ-1 as two candidate NRF2 network modulators for treating trigeminal neuropathic pain. We find that exemestane induces the NRF2 network through NRF2 itself, whereas JQ-1 recruits the network differently. In contrast to current pharmacologic agents that mask pain by blunting nerve firing, increasing the NRF2 transcriptional network may be a therapeutic approach that seeks to improve pain through redox control.

A few patients on systemic exemestane report musculoskeletal stiffness. Recently, Fusi *et al.* (*94*) rigorously examined whether exemestane can elicit these symptoms through TRPA1. They find that exemestane can elicit acute allodynia in mice but within 3 hours of dosing. We observe that at the dose of 10 mg/kg used in many preclinical models (*95*, *96*), exemestane exerts a powerful analgesic effect that persists over the course of days (Fig. 7, A and B). If normalizing dose to body surface area through allometric scaling, a mouse dose of 10 mg/kg is equivalent to 0.8 mg/kg in humans or 48 mg for a 60-kg person. This dose is well within the 25- to 50-mg dosing used clinically. In addition, the maximum serum concentration that exemestane reaches in patients falls between 0.05 and 0.1 μM (*97*, *98*), 500- to 1000-fold lower than the EC_50_ value at which exemestane activates TRPA1 in vitro. Thus, we propose that clinically relevant concentrations of exemestane may not activate TRPA1 but that supratherapeutic concentrations might and thus should be avoided when treating patients (fig. S12, A and B). We also note that directly applying exemestane to the trigeminal nerve is also analgesic in mice (Fig. 7, F and G), suggesting that percutaneous, stereotactic administration of exemestane to the trigeminal nerve in humans may be a safe and feasible approach to treating trigeminal neuralgia while minimizing the potential adverse effects of systemic exemestane (*94*). Further drug discovery could alternatively refine exemestane or identify new exemestane-like agents to minimize potential adverse effects. In contrast, neither sulforaphane nor JQ-1 activates TRPA1 (fig. S12, C and D).

While our findings suggest that elevated oxidative stress contributes to trigeminal neuropathic pain in both patients and mice, they do not exclude other mechanisms. Recently, Trevisan *et al.* (*29*) found that depleting monocytes and macrophages with clodronate or an antibody against the chemoattractant chemokine ligand 2 lessens allodynia in a mouse model of trigeminal neuralgia. Their findings demonstrate that inflammation contributes to trigeminal neuralgia by eliciting redox stress. In this same vein, the Kultima group found that inflammatory biomarkers are elevated in the CSF from patients with trigeminal neuralgia (*99*, *100*). Paracrine signaling by TRPA1 in non-neural cells may also promote neuroinflammation (*101*). In addition, TRPA1 may be recruited to the membrane (*102*) or acutely sensitized (*103*) during episodes of pain. Neuronally, aberrant ephaptic coupling (*104*) and afferent circuit plasticity (*105*) may also be interpreted as pain.

A final note is that the preclinical model used in this and other studies involves constriction of the maxillary segment of the trigeminal nerve, which is a location distinct from the cisternal segment that is compressed by the superior cerebellar artery in most patients with typical trigeminal neuralgia. An improved model would involve the intradural compression of the preganglionic trigeminal nerve, although the feasibility of this study in mice is questionable. Whether this commonly used preclinical model sheds mechanistic insight into typical trigeminal neuralgia remains to be fully proven (*106*). Nevertheless, our data reveal that targeting the NRF2 transcriptional network alone is analgesic, suggesting that repurposing NRF2 transcriptional network modulators may be a mechanistically alternative approach toward managing trigeminal neuropathic pain.

## MATERIALS AND METHODS

### Experimental model and subject details

#### Human

##### Institutional review board approval

Patients with trigeminal neuralgia, Chiari malformations, idiopathic normal pressure hydrocephalus, or pseudotumor cerebri were recruited under protocols approved by the Institutional Review Board at the Johns Hopkins University School of Medicine (study numbers IRB00103861 and NA_00029413).

##### Cerebrospinal fluid

CSF from patients was collected into specimen tubes, transferred to 15-ml conical tubes, and subsequently centrifuged for 5 min at 2000*g* within 2 hours of collection. The supernatant was then collected, aliquoted, and stored at −80°C until experimentation.

#### Mice

##### Animal care and use

All experiments were performed in accordance with protocols approved by the Animal Care and Use Committee at the Johns Hopkins University School of Medicine.

##### WT mice

C57BL/6J (stock no. 000664) and B6129PF2/J (stock no. 100903) WT mice were purchased from the Jackson Laboratory.

##### TRPA1 knockout (TRPA1^−/−^) mice

TRPA1^−/−^ mice were generated as previously described (*35*) and purchased from the Jackson Laboratory (stock no. 006401).

##### Keap1(f/f) mice

*Keap1*(*f*/*f*) mice were generated as previously described (*107*). Tamoxifen-inducible *Keap1*(*f*/*f*)/CMV-CreER mice were generated by crossing *Keap1*(*f*/*f*) mice with CAG-CreER^+^ mice. To induce excision of *Keap1*, *Keap1*(*f*/*f*)/CMV-CreER was injected intraperitoneally with tamoxifen (75 mg/kg) once every 24 hours over five consecutive days. *Keap1*(*f*/*f*) (*Cre*-negative) mice were similarly injected with tamoxifen and served as controls (Fig. 4C). Disruption of *Keap1* was confirmed by genomic polymerase chain reaction (PCR) using primers listed in Table 1 (Key resources table) and by quantitative RNA PCR for *Keap1* (Fig. 4, D and E). Nrf2 activation was confirmed by measuring the expression of the canonical target NADPH (reduced form of nicotinamide adenine dinucleotide phosphate) quinone oxidoreductase 1 (*Nqo1*) by quantitative RNA PCR (Fig. 4F). Behavioral and molecular tests were only performed seven or more days after the final tamoxifen injection.

**Table 1.** Key resources table. N/A, not available.

**Reagent or resource**	**Source**	**Identifier**
**Antibodies**		
Rabbit anti–4-hydroxynonenal	Abcam	Cat# ab46545; RRID:AB_722490
Rabbit anti-TRPA1	Novus Biologicals	Cat# NB110-40763; RRID:AB_715124
Rabbit anti-DNP	Millipore Sigma	Cat# 90451
Rabbit anti-NRF2	Cell Signaling	Cat# 12721; RRID: AB_2715528
Chicken anti-MAP2	Abcam	Cat# ab5392; RRID: AB_2138153
Chicken anti-MBP	Millipore Sigma	Cat# AB9348; RRID: AB_11213157
Mouse anti–β-actin (HRP-conjugated)	Santa Cruz Biotechnology	Cat# sc-47778 HRP; RRID: AB_2714189
Donkey anti-rabbit IgG (HRP-conjugated)	GE Healthcare	Cat# NA934; RRID: AB_772206
Goat anti-rabbit IgG (HRP-conjugated)	Millipore Sigma	Cat# 90452
Goat anti-rabbit IgG (Alexa Fluor 568–conjugated)	Invitrogen	Cat# A-11011; RRID: AB_143157
Goat anti-chicken IgG (Alexa Fluor 488–conjugated)	Invitrogen	Cat# A-11039; RRID: AB_142924
Rat anti-HA	Roche	Cat# ROAHAHA; RRID: AB_2687407
Mouse anti-myc	Millipore Sigma	Cat# M4439; RRID: AB_439694
Mouse anti-FLAG	Millipore Sigma	Cat# F1804; RRID: AB_262044
Rat anti-mouse IgG for IP (HRP-conjugated)	Abcam	Cat# ab131368; RRID: AB_2895114
Goat anti-rat IgG (HRP-conjugated)	R&D Systems	Cat# HAF005; RRID: AB_1512258
Horse anti-mouse IgG (HRP-conjugated)	Cell Signaling	Cat# 7076; RRID: AB_330924
**Biological samples**		
Human cerebrospinal fluid (patients with trigeminal neuralgia)	This paper	Johns Hopkins University School of Medicine IRB# 00103861
Human cerebrospinal fluid (patients with idiopathic normal pressure hydrocephalus)	This paper	Johns Hopkins University School of Medicine IRB# NA_00029413
Human cerebrospinal fluid (patients with pseudotumor cerebri)	This paper	Johns Hopkins University School of Medicine IRB# NA_00029413
Human cerebrospinal fluid (patients with Chiari malformations)	This paper	Johns Hopkins University School of Medicine IRB# 00103861
**Chemicals, peptides, and recombinant proteins**		
dl-Sulforaphane	Sigma-Aldrich	Cat# S4441
Exemestane	Tocris	Cat# 3759
Letrozole	Cayman	Cat# 11568
(+)-JQ-1	MedChemExpress	Cat# HY-13030
(−)-JQ-1	MedChemExpress	Cat# HY-13030A
Tamoxifen	Sigma-Aldrich	Cat# T5648
(Z)-4-hydroxytamoxifen (4-OHT)	Sigma-Aldrich	Cat# H7904
Fluo-4 AM	Invitrogen	Cat# F14201
Fura-2 AM	Invitrogen	Cat# F1221
(+)-Sodium l-ascorbate	Sigma-Aldrich	Cat# A7631
AM-0902	Tocris	Cat# 5914
Olive oil	Sigma-Aldrich	Cat# O1514
Corn oil	Sigma-Aldrich	Cat# C8267
Dulbecco’s modified Eagle’s medium	Gibco	Cat# 11960044
Fetal bovine serum	Sigma-Aldrich	Cat# F2442
Penicillin/streptomycin	Gibco	Cat# 15140122
l-Glutamine	Gibco	Cat# A2916801
Collagenase/dispase	Sigma-Aldrich	Cat# 10269638001
Hanks’ balanced salt solution (HBSS)	Gibco	Cat# 14025076
Laminin	Roche	Cat# 11243217001
Poly-d-lysine hydrobromide	Sigma-Aldrich	Cat# P6407
Protease inhibitor cocktail	Sigma-Aldrich	Cat# P8340
Normal goat serum (10%)	Thermo Fisher Scientific	Cat# 50062Z
EZview Red Anti–c-Myc Affinity Gel	Millipore Sigma	Cat# E6654
Lipofectamine 3000 Transfection Reagent	Thermo Fisher Scientific	Cat# L3000001
cOmplete Mini Protease Inhibitor Cocktail	Roche	Cat# 11836153001
**Critical commercial assays**		
OxyBlot Protein Oxidation Detection Kit	Millipore Sigma	Cat# S7150
TBARS (Lipid Peroxidation) Assay	Cell Biolabs Inc.	Cat# STA-330
Platinum Taq DNA Polymerase High Fidelity Kit	Invitrogen	Cat# 11304011
TaqMan RNA-to-Ct 1-Step Kit	Applied Biosystems	Cat# 4392656
DNeasy Blood & Tissue Kit	Qiagen	Cat# 69504
RNeasy Plus Universal Kit	Qiagen	Cat# 73404
Human Hemoglobin ELISA Kit	Invitrogen	Cat# EH237RB
Luciferase Assay System	Promega	Cat# E1500
**Deposited data**		
RNA sequencing from fibroblasts treated with 0.25 μM JQ-1	Shin *et al.* (*85*)	Gene Expression Omnibus (GEO) accession no. GSE130313
**Experimental models: Cell lines**		
Primary neurons from WT mice	This paper	N/A
Primary neurons from TRPA1^−/−^ mice	This paper	N/A
Human Embryonic Kidney (HEK) 293 cells	American Type Culture Collection	Cat# CRL-1573; RRID: CVCL_0045
NRF2/ARE Luciferase Reporter HEK-293 Stable Cell Line	Signosis Inc.	Cat# SL-0042-NP
**Experimental models: Organisms/strains**		
Mouse: WT (C57BL/6J)	The Jackson Laboratory	Cat# 000664
Mouse: WT (B6129PF2/J)	The Jackson Laboratory	Cat# 100903
Mouse: TRPA1^−/−^ (B6;129P-Trpa1^tm1Kykw^/J)	The Jackson Laboratory	Cat# 006401
Mouse: Keap1(*f*/*f*)	Blake *et al.* (*107*)	N/A
Mouse: Keap1(*f*/*f*)/CMV-CreER	Sussan *et al.* (*117*)	N/A
Mouse: NRF2^−/−^ (Nfe2l2^tm1Ywk^/J)	The Jackson Laboratory; Chan *et al.* (*108*)	Cat# 017009
**Oligonucleotides**		
Forward primer sequence used for genotyping Keap1(*f*/*f*) mice	CGAGGAAGCGTTTGCTTTAC	N/A
Reverse primer sequence used for genotyping Keap1(*f*/*f*) mice	GAGTCACCGTAAGCCTGGTC	N/A
Forward primer sequence used for cloning NRF2 constructs: Sal I hNRF2 5’ Fwd	TCGGTCGACAATGATGGACTTGGAGCTGCCGCCGC	N/A
Reverse primer sequence used for cloning NRF2 constructs: Xho I hNRF2 3’ Rev	AACCTCGAGTTAGTTTTTCTTAACATCTGGCT TCTTACTTTTGGGA	N/A
**Recombinant DNA**		
Plasmid: myc-TRPA1 (human)	Macpherson *et al.* (*49*)	N/A
Plasmid: myc-NRF2 (human)	This paper	N/A
Plasmid: FLAG-KEAP1 (human)	Fan *et al.* (*118*)	Addgene plasmid # 28023; RRID: Addgene_28023
Plasmid: HA-Ubiquitin (human)	Kamitani *et al.* (*119*)	Addgene plasmid # 18712; RRID: Addgene_18712
**Software and algorithms**		
GraphPad Prism	GraphPad Software Inc	graphpad.com
FIJI	NIH	imagej.net
BioJupies	Torre *et al.* (*115*)	amp.pharm.mssm.edu/biojupies/
Connectivity Map	Lamb *et al.* (*120*); Subramanian *et al.* (*76*)	clue.io/cmap

##### NRF2 knockout (NRF2^−/−^) mice

NRF2^−/−^ mice were generated as previously described (*108*) and purchased from the Jackson Laboratory (stock no. 017009).

### Method details

#### Materials and preparation

##### Compounds

Compounds were obtained as follows: sulforaphane (Sigma-Aldrich), exemestane (Tocris), letrozole (Cayman), (+)-JQ-1 and (−)-JQ-1 (MedChemExpress), tamoxifen (Sigma-Aldrich), 4-OHT (Sigma-Aldrich), hematoxylin (Sigma-Aldrich), eosin Y (Sigma-Aldrich), osmium tetroxide (Sigma-Aldrich), Fluo-4 AM (Invitrogen), Fura-2 AM (Invitrogen), ascorbate (Sigma-Aldrich), AM-0902 (Tocris), olive oil (Sigma-Aldrich), corn oil (Sigma-Aldrich), Dulbecco’s modified Eagle’s medium (DMEM; Gibco), fetal bovine serum (Sigma-Aldrich), penicillin-streptomycin (Gibco), l-glutamine (Gibco), forskolin (Sigma-Aldrich), collagenase/dispase (Sigma-Aldrich), Hanks’ balanced salt solution (HBSS; Gibco), laminin (Roche), poly-d-lysine (Sigma-Aldrich), protease inhibitor cocktail (Sigma-Aldrich), and 10% normal goat serum (Thermo Fisher Scientific).

##### Material preparation

All drugs were freshly prepared in an appropriate solvent immediately before beginning each treatment course. Each drug was then aliquoted into individual tubes for each day and stored at −20°C before thawing at 4°C just before treatment. Sulforaphane was dissolved in 100% dimethyl sulfoxide (DMSO) and then diluted to 1% DMSO in saline. Exemestane, letrozole, (+)-JQ-1, and (−)-JQ-1 were dissolved in 100% DMSO and then diluted to 5% DMSO in corn oil. Ascorbate was dissolved directly in saline. AM-0902 was dissolved in 100% DMSO and then diluted to 5% DMSO in olive oil. Tamoxifen was dissolved directly in corn oil. All other compounds were prepared as 50- to 1000-μl aliquots and stored at −20°C before thawing at 4°C. Freeze/thaw cycles were avoided whenever possible.

#### Plasmids/cDNA

Complementary DNA (cDNA) encoding myc-TRPA1 was a gift from M. Caterina (Johns Hopkins University, USA).

#### Constriction of the maxillary nerve

Constriction of the maxillary division of the trigeminal nerve was performed as previously described (*40*, *41*). The procedure was performed under direct visualization and control with a surgical microscope. Mice were first anesthetized with ketamine [100 mg/kg, intraperitoneally (i.p.)] and xylazine (12.5 mg/kg, i.p.) and monitored by pinching the skin between the toes with forceps and monitoring for withdrawal. Mice were restrained with adhesive tape to a sterilized polystyrene board. Upon sufficient anesthesia, the scalp was shaved, and an anterior-to-posterior skin incision was made at the midline to expose the nasal and maxillary bones. The maxillary nerve was exposed and carefully dissected free from the surrounding connective tissue. The distal end of the maxillary nerve was then loosely constricted using 8-0 silk sutures as a ligature. The sutures were tied using a slip knot followed by a normal knot, after which any remaining suture was cut free. The incision was then closed with a 4-0 silk suture. In the sham procedure, the left maxillary nerve was exposed but not constricted. Mice were monitored and rehydrated until fully recovered from anesthesia.

#### Pharmacologic treatments

##### Sulforaphane, exemestane, and JQ-1

Mice were dosed with either sulforaphane, exemestane, letrozole, (+)-JQ-1, (−)-JQ-1, or ascorbate as indicated below. Mice were first dosed daily for 2 days before surgery and again daily just after behavior testing with the following: sulforaphane (10 mg/kg, i.p.), exemestane (10 mg/kg, i.p.), letrozole (10 mg/kg, i.p.), (+)-JQ-1 (40 mg/kg, i.p.), (−)-JQ-1 (40 mg/kg, i.p.), and ascorbate (100 mg/kg, i.p.).

##### Local exemestane

Exemestane was applied directly to the maxillary nerve as indicated below. Mice received a single dose during surgery [exemestane (5 μl, 25 μg total)].

##### AM-0902

Mice were dosed with AM-0902 as indicated below. Mice treated with AM-0902 were dosed 30 min before behavior testing [AM-0902 (30 mg/kg, p.o.)].

##### Tamoxifen

Mice were dosed with tamoxifen as indicated below. Mice were dosed once every 24 hours over five consecutive days. Behavioral and molecular tests were only performed seven or more days after the final tamoxifen injection [tamoxifen (75 mg/kg, i.p.)].

#### Pain behavior assays

##### Mechanical allodynia

Mechanical allodynia was assessed in C57BL/6J, TRPA1^+/+^, TRPA1^−/−^, *Keap1*(*f*/*f*), and *Keap1*(*f*/*f*)/CMV-CreER mice using a Von Frey filament as previously outlined (*40*, *41*). Animals were placed individually in transparent plastic boxes and allowed to acclimate to the environment for at least 30 min before testing. After habituation, a 0.04–*g* force von Frey filament was used to stimulate the territory innervated by the maxillary nerve, including the vibrissal skin pad. Each mouse’s response to the filament was scored from 0 to 4 on the scale below. Each mouse was scored 10 times per day. Changes in mechanical allodynia were considered relative to sham- or vehicle-treated animal controls. Behavior was assessed concomitantly or in a blocked manner with consideration for both genotype and treatment as follows: 0, no response; 1, nondefensive response to the stimulus (i.e., mouse nondefensively explores the filament); 2, withdrawal response (i.e., mouse turns its head away from the filament); 3, escape/attack response (i.e., mouse moves its body away from the filament and assumes crouching position against the box wall; actively attacks the filament by biting and/or grabbing); 4, asymmetric face grooming (i.e., mouse wipes the stimulated facial area in an uninterrupted series of at least three face-wash strokes).

##### Cold allodynia

Cold allodynia was assessed in C57BL/6J, TRPA1^+/+^, TRPA1^−/−^, *Keap1*(*f*/*f*), and *Keap1*(*f*/*f*)/CMV-CreER mice using ice-cold acetone as previously outlined (*109*). Animals were placed individually in transparent plastic boxes and allowed to acclimatize to the environment for at least 30 min before testing. After habituation, 20 μl of cold acetone was applied to the ligated vibrissal pad skin surface. Cold allodynia was measured as the average time spent wiping the region in a 60-s period, allowing for a lull of 5 s between bouts of wiping. Allodynia was measured three times with 10 min between intervals. Changes in cold allodynia were considered relative to sham- or vehicle-treated animal controls. Behavior was assessed concomitantly or in a blocked manner with consideration for both genotype and treatment.

#### Immunoblotting

##### Western blotting

Western blotting was performed as previously described (*110*, *111*). Briefly, tissues were homogenized at 4°C in lysis buffer [solution of 50 mM tris-HCl, 150 mM NaCl, 0.1% SDS, 0.5% sodium deoxycholate, and 1% Triton X-100 (pH 7.4)] supplemented with protease inhibitors (Sigma-Aldrich). When dissecting maxillary nerves for immunoblot analysis, nerves were dissected both as proximally toward the semilunar ganglion and as distal to the skin as possible. Nerves were harvested 11 days after surgery, immediately after the final behavioral test. Lysates were then pulse-sonicated and centrifuged at 16,000*g* for 10 min at 4°C. Fifteen micrograms of cleared lysate was run on a 4 to 12% polyacrylamide Bis-Tris gradient gel in running buffer [solution of 50 mM MES, 50 mM tris base, 0.1% SDS, and 1 mM EDTA (pH 7.3)] and then transferred to a polyvinylidene difluoride membrane. Membranes were blocked with 5% milk in TBS-T [solution of 16 mM tris-HCl, 140 mM NaCl, and 0.1% Tween 20 (pH 7.6)] for 1 hour at 25°C and then incubated with primary antibodies in 3% bovine serum albumin (BSA) (w/v) in TBS-T overnight at 4°C. The following day, membranes were washed with TBS-T and then incubated with secondary antibodies in 3% BSA (w/v) in TBS-T for 1 hour at 25°C. The following primary antibodies were used: rabbit anti–4-HNE (1:1000; Abcam, ab46545), rabbit anti-TRPA1 (1:1000; Novus Biologicals, NB110-40763), rabbit anti-2,4-dinitrophenol (1:150; Millipore Sigma, 90451), and mouse anti–β-actin [1:10,000; Santa Cruz Biotechnology, sc-47778 horseradish peroxidase (HRP)]. The following secondary antibodies were used: donkey anti-rabbit immunoglobulin G (IgG) (1:10,000; GE Healthcare, NA934) and goat anti-rabbit (1:300; Millipore Sigma, 90452). Immunoblots were quantified by densitometry as described below in the “Quantification and statistical analysis” section.

##### Dot blotting

Ten microliters of each CSF sample was spotted onto a nitrocellulose membrane in a dot blot manifold and vacuum-dried. Membranes were then blocked with 3% BSA (w/v) in TBS-T for 1 hour at 25°C and then incubated with a rabbit anti–4-HNE (1:1000; Abcam, ab46545) primary antibody in 3% BSA (w/v) overnight at 4°C. The following day, membranes were washed with TBS-T and then incubated with a donkey anti-rabbit IgG (1:10,000; GE Healthcare, NA934) secondary antibody in 3% BSA for 1 hour at 25°C.

#### Oxidative stress assays

##### 4-Hydroxynonenal

4-HNE was quantified by immunoblot as described above in the “Western blotting” section with a rabbit anti–4-HNE (1:1000; Abcam, ab46545) primary antibody.

##### Protein carbonylation

Protein carbonylation was assessed with the OxyBlot Protein Oxidation Detection Kit (listed in Table 1) as per the manufacturer’s instructions. Protein carbonylation was quantified by immunoblot as described above in the “Western blotting” section with a rabbit anti-DNP (1:150; Millipore Sigma, 90451) primary antibody.

##### Malondialdehyde

MDA was quantified with the OxiSelect TBARS Assay Kit (listed in Table 1) as per the manufacturer’s instructions. Concentrations below the limit of detection (LOD) were censored and substituted with a constant value of the LOD/√2 (*112*).

#### Hemoglobin ELISA

CSF hemoglobin was quantified with the Human Hemoglobin ELISA (enzyme-linked immunosorbent assay) Kit (listed in Table 1) as per the manufacturer’s instructions. Concentrations below the LOD were censored and substituted with a constant value of the LOD/√2 (*112*).

#### Cell culture and transfection

##### HEK-293 cells

HEK-293 cells were cultured in DMEM, 10% fetal bovine serum, penicillin/streptomycin (100 U/ml), and glutamine (2 mM) in an atmosphere of 5% CO_2_ at 37°C as previously described (*111*).

##### Trigeminal ganglia dissociation and culture

Trigeminal ganglia were pooled into cold DH10 media [90% DMEM, 10% fetal bovine serum, and penicillin/streptomycin (100 U/ml)]. Trigeminal ganglia were digested with dispase (5 mg/ml)/collagenase (1 mg/ml) in HBSS at 37°C for 30 to 45 min. Cells were then triturated, pelleted at 1000*g* for 5 min at 25°C, gently rinsed twice in DH10, and then resuspended in DH10. Dissociated cells were then plated onto glass coverslips coated with poly-d-lysine (0.5 mg/ml) and laminin (10 μg/ml). Neurons were cultured in DH10 supplemented with nerve growth factor (50 ng/ml) at 37°C for 12 to 16 hours.

##### Transfection

Transfection was performed as previously described (*111*). HEK-293 cells were transiently transfected with Lipofectamine 3000 (Thermo Fisher Scientific) transfection reagents according to the manufacturer’s instructions. Cells were harvested 24 to 48 hours after transfection for downstream analysis.

#### Immunoprecipitation

Immunopreciptation was performed as previously described with a few modifications (*111*). Briefly, cells were harvested on ice in lysis buffer [50 mM tris-HCl, 150 mM NaCl, 1% Triton X-100, and 5% glycerol (v/v) (pH 7.6 solution)] supplemented with protease inhibitors and centrifuged at 16,000*g* for 10 min at 4°C. Anti–c-Myc affinity gel (Millipore Sigma) was added to cleared lysates and rotated overnight at 4°C. Five percent of each sample was set aside as input before addition of beads. The following day, beads were pelleted by centrifugation at 1000*g* for 30 s at 4°C. The supernatant was aspirated, and beads were washed five times in lysis buffer by repeat centrifugation, aspiration, and resuspension. Proteins were eluted from beads by boiling for 5 min in lithium dodecyl sulfate sample buffer (Thermo Fisher Scientific), and the eluates were analyzed by immunoblot as described above.

#### Subcellular fractionation

Subcellular (nuclear versus cytoplasmic) fractionation was performed as previously described with a few modifications (*113*). Briefly, HEK-293 cells transiently transfected with the proteins of interest were lysed and snap-frozen in subcellular lysis buffer [10 mM Hepes, 1 mM EGTA, 1 mM dithiothreitol, and 10% sucrose (w/v) (pH 7.4 solution)] supplemented with protease inhibitors. The lysates were than thawed and filtered through a 70-μm nylon filter to remove intact cells. An aliquot of the thawed filtered lysate was retained as the whole-cell fraction, and the remaining lysate was centrifuged at 1000*g* for 10 min at 4°C. The resulting supernatant was retained as the crude membrane/cytoplasmic fraction, and the pellet was washed two times in lysis buffer by repeat resuspension, centrifugation, aspiration, and resuspension. The pellet was then resuspended in lysis buffer and retained as the nuclear fraction. The membrane/cytoplasmic fraction was then centrifuged at 21,000*g* for 20 min at 4°C, and the resulting supernatant was retained as the cytoplasmic fraction. The whole-cell, cytoplasmic, and nuclear fractions were then sonicated on ice and clarified one last time at 16,000*g* for 10 min at 4°C. The fractions were then analyzed by immunoblot as described above.

#### Calcium imaging and analysis

Calcium imaging and analysis were performed as previously described (*114*). Briefly, cells were imaged in calcium imaging buffer [CIB; 10 mM Hepes, 1.2 mM NaHCO_3_, 130 mM NaCl, 3 mM KCl, 2.5 mM CaCl_2_, 0.6 mM MgCl_2_, 20 mM glucose, and 20 mM sucrose (at pH 7.4 and 290 to 300 mosM)]. To monitor changes in intracellular [Ca^2+^] ([Ca^2+^]_i_), cells were loaded with either 1 μM Fura 2-AM (HEK-293 cells) or 1 μM Fluo 4-AM (trigeminal neurons) for 30 min in the dark at 37°C in CIB just before imaging. With Fura 2-AM, emission at 510 nm was monitored following excitation at both 340 and 380 nm. With Fluo 4-AM, emission at 520 nm was monitored following excitation at 488 nm. Cells were identified as positively responding if [Ca^2+^]_i_ rose by 15% compared to baseline. Damaged, detached, high-baseline, and motion-activated cells were excluded from analysis.

##### HEK-293 cells

HEK-293 cells were plated on poly-d-lysine–coated coverslips and transiently transfected with the vector backbone or constructs encoding WT or mutant TRPA1. Unless otherwise noted, cells were imaged for 30 s to establish a baseline before compounds were added. Vehicle was first applied for 30 s, after which 100 μM iodoacetamide, 1 mM H_2_O_2_, or 100 μM 4-HNE was applied. CSF from cases and controls was diluted into CIB 1:50 before each trial. Fifty micromolar of the nonselective TRP channel inhibitor ruthenium red was applied at the end of every imaging trial.

##### Trigeminal ganglia

Neurons were incubated with Fluo-4 AM 12 to 16 hours after dissociation. Unless otherwise noted, neurons were imaged for 30 s to establish a baseline before compounds were added. Vehicle was first applied for 30 s, after which CSF was applied for 60 s and then 100 nM capsaicin for 30 s. Randomly pooled CSF from cases or controls was diluted into CIB 1:1 before each trial. At the end of every imaging trial, 50 mM KCl was added as a positive control. Percentage activated was determined as described earlier. To distinguish pain- and itch-encoding neurons from other neurons in the culture, we filtered our analysis to neurons that also responded to the TRPV1 agonist capsaicin (*53*). Previous studies have demonstrated that TRPA1 and TRPV1 are coexpressed in pain- and itch-encoding sensory neurons, and either can serve to unbiasedly identify such neurons (*42*, *48*).

#### NRF2/ARE luciferase reporter assay

NRF2 activity was monitored with a reporter cell line in which changes in NRF2 activity are coupled to the expression of firefly luciferase (NRF2/ARE Luciferase Reporter HEK-293 Stable Cell Line, listed in Table 1). Luciferase activity was quantified with the Luciferase Assay System (listed in Table 1) as per the manufacturer’s instructions. Briefly, cells were plated at 125,000 cells per well in a 24-well plate. The following day, cells were treated with either vehicle or varying doses of sulforaphane, exemestane, or JQ-1 for 6 hours. Cells were then lysed in passive lysis buffer (Promega, E1941) supplemented with protease inhibitors (Sigma-Aldrich). Lysates were then cleared at 16,000*g* for 15 min at 4°C. Fifteen microliters of each lysate was mixed with 5 μl of luciferase assay substrate (Promega). Total light was measured using a luminometer with a 10-s integration time with a delay of 2 s. Luciferase measurements were normalized to total protein and reported as normalized to the vehicle condition.

#### Histology

Maxillary nerves from mice were dissected and fixed in cold 4% formaldehyde (v/v) overnight at 4°C. Tissues were then cryoprotected through a series of 10, 20, and 30% sucrose (w/v) gradients for 24 hours each at 4°C. Tissues were then embedded in Optimal Cutting Temperature compound and sectioned in 20-μm intervals with a cryostat, after which the sections were dried onto slides and kept at −20°C. Sections were then processed for immunohistochemistry.

Immunohistochemistry was performed as previously described (*110*). Briefly, sections were postfixed with 4% paraformaldehyde for 15 min at 25°C and then permeabilized with 100% methanol for 7 min at −20°C. The slides were then preincubated in blocking solution (10% normal goat serum, Thermo Fisher Scientific) for 30 min at 25°C. Sections were incubated overnight at 4°C with the appropriate primary antibodies (below) in blocking solution. Sections were washed and incubated with the appropriate secondary antibodies (below) diluted 1:250 in blocking solution for 1 hour at 25°C. Tissues were then mounted with ProLong Gold Antifade Mountant with 4′,6-diamidino-2-phenylindole (DAPI) (Invitrogen).

#### NRF2/MAP2 immunostaining

Mice were first dosed daily with either sulforaphane (10 mg/kg, i.p.), exemestane (10 mg/kg, i.p.), or the appropriate vehicle for 2 days before surgical constriction of the maxillary nerve, after which they were dosed every 24 hours thereafter. NRF2 immunostaining was performed 24 hours after the fourth dose. The following antibodies were used: primary antibodies: rabbit anti-NRF2 (1:100) and chicken anti-MAP2 (1:5000); secondary antibodies: goat anti-rabbit (Alexa 568, A-11011, Invitrogen) and goat anti-chicken (Alexa 488, A-11039, Invitrogen).

#### RNA sequencing and analysis

##### Sequencing

RNA sequencing was performed as previously described (*85*). To assess the transcriptional signature of JQ-1, total RNA was isolated from three independent human fibroblast lines after 48 hours of treatment with DMSO or 0.25 μM JQ-1 using TRIzol and RNeasy isolation columns (Qiagen) as per the manufacturer’s instructions. DNA was digested with deoxyribonuclease treatment. All samples had RNA integrity numbers 9.60 or higher as measured with an Agilent 2100 Bioanalyzer. mRNA was enriched by polyadenylate selection, prepped using an Illumina TruSeq mRNA sample preparation kit, and sequenced by Illumina HiSeq 2000.

##### Differential gene expression and pathway enrichment analyses

Differential gene expression analysis was performed as previously described (*111*) using BioJupies (*115*). We leveraged BioJupies to perform Pathway Enrichment Analyses through Enrichr (*116*) to identify the biological processes that are overrepresented in the gene set up-regulated by JQ-1 treatment.

#### DNA/RNA isolation, PCR, and quantitative PCR

Total cellular or tissue DNA/RNA was extracted using the DNeasy Blood & Tissue Kit (Qiagen) for DNA or the RNeasy Plus Universal Kit (Qiagen) for RNA as per the manufacturer’s instructions, as previously described (*111*). PCR was performed with the Platinum Taq DNA Polymerase High Fidelity Kit (Invitrogen), whereas qPCR was performed with the TaqMan RNA-to-Ct 1-Step Kit (Applied Biosystems). All reagents are listed in Table 1.

#### In silico candidate drug screening

We queried the CMAP (clue.io) to identify molecules most likely to reproduce the transcriptional signature of both overexpressing *Nfe2l2* (the gene encoding NRF2) and genetically silencing *Keap1*. CMAP scores how well each compound’s transcriptional profile matches the query signature from −100 to +100, with a score of +100 indicating a complete match. Molecules with higher scores were interpreted as most likely mimicking up-regulating the NRF2 transcriptional network. In the *Nfe2l2*-queried signature, 187 compounds receive a Connectivity Score above CMAP’s recommended cutoff of +90, whereas 114 compounds score above +90 in the *Keap1*-derived signature. Exemestane and JQ-1 were two overlapping candidate compounds with high scores in both the *Nfe2l2*-derived and *Keap1*-derived signatures and were thus prioritized for further validation. Exemestane scored 99.5772 in the *Nfe2l2*-derived signature and 95.587 in the *Keap1*-derived signature. JQ-1 scored 99.3935 in the *Nfe2l2*-derived signature and 99.7503 in the *Keap1*-derived signature.

### Quantification and statistical analysis

Quantification and statistical analysis were performed as previously described (*111*). All data were plotted and expressed as the median and range, means ± SEM, or means ± 95% CI, as noted. Statistical comparisons were performed using two-tailed unpaired Student’s *t* tests, Fisher’s exact tests, or analysis of variance (ANOVA) analyses, as noted with tests correcting for multiple testing as appropriate. Differences were considered significant at *P* < 0.05.

For densitometry analyses of immunoblots, background was subtracted with a rolling ball radius of 50.0 pixels and/or by measuring signal at an empty segment of the membrane. Protein carbonylation levels were quantified by averaging the densitometry of each lane from the highest to lowest molecular weights at which signal was 10 U above background and then normalizing to β-actin. 4-HNE levels were quantified by summating densitometries of individual regions within lanes at which signal was 10 U above background and then normalizing to β-actin.

All in vivo experiments were performed concomitantly or in a blocked manner with consideration for both genotype and treatment. Mice were excluded from behavioral analysis if they displayed signs of vehicle or drug toxicity or severe, unrelenting pain.

## Notes

View/request a protocol for this paper from *Bio-protocol*.
