# Supplementary material for: Identification of the NRF2 transcriptional network as a therapeutic target for trigeminal neuropathic pain
Source: Sci Adv. 2022 Aug 3;8(31):eabo5633. doi: 10.1126/sciadv.abo5633 (PMC9348805; doi:10.1126/sciadv.abo5633)
Supplement: Supplementary file 1 — Figs. S1 to S12 [file sciadv.abo5633_sm.pdf]

Science Advances
AAAS

Supplementary Materials for

Identification of the NRF2 transcriptional network as a therapeutic target for trigeminal neuropathic pain

Chirag Vasavda *et al.*

Corresponding author: Solomon H. Snyder, ssnyder@jhmi.edu; Michael Lim, mklim@stanford.edu

Sci. Adv. **8**, eabo5633 (2022)
DOI: 10.1126/sciadv.abo5633

The PDF file includes:

Figs. S1 to S12
Legends for tables S1 to S3

Other Supplementary Material for this manuscript includes the following:

Tables S1 to S3

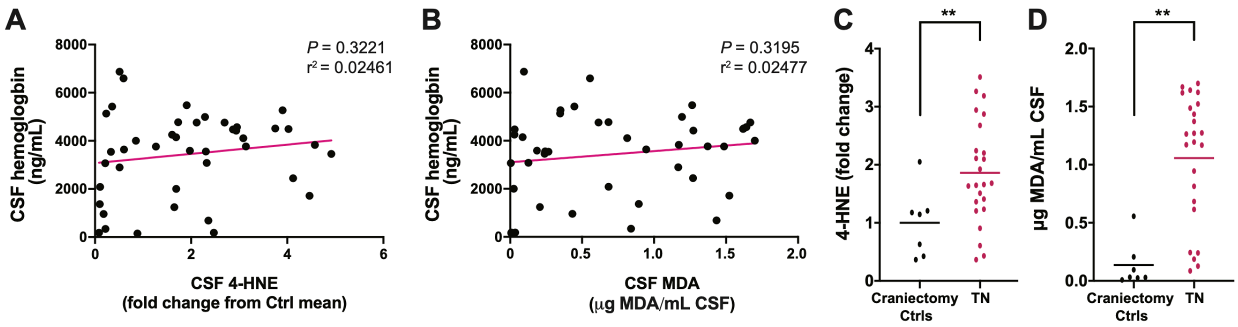

Figure S1. CSF reactive oxygen species do not correlate CSF hemoglobin or surgery.
A-B, Relationship between cerebrospinal fluid (CSF) hemoglobin and (*A*) CSF 4-hydroxynonenal (4-HNE) and (*B*) CSF malondialdehyde (MDA). Points represent individual patients. **C**, Comparison of relative 4-HNE in CSF from patients with trigeminal neuralgia (TN) normalized to average 4-HNE in CSF from patients who underwent posterior fossa craniectomies (Craniectomy Ctrls). Points represent individual patients. **D**, Comparison of normalized MDA (μg MDA/mL CSF) in CSF from patients with trigeminal neuralgia (TN) and CSF from patients who underwent posterior fossa craniectomies (Craniectomy Ctrls). Points represent individual patients. (*C*-*D*) Mean depicted. ** = $P < 0.01$ by two-tailed unpaired Student's t-test.

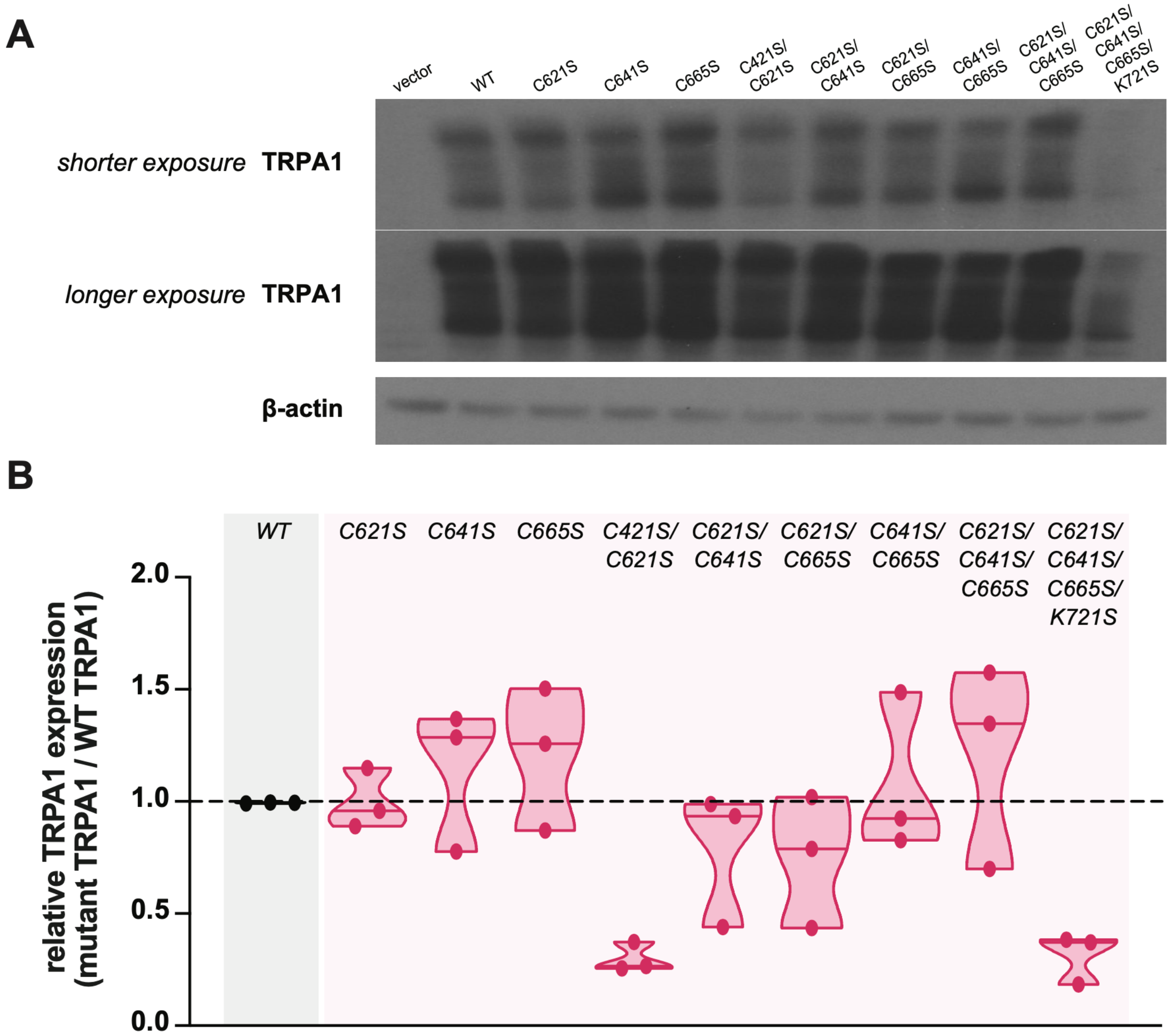

Figure S2. Expression of WT and targeted TRPA1 cysteine/lysine mutants.

A, Representative immunoblot for WT and targeted TRPA1 cysteine/lysine mutants and β-actin. *above*, shorter exposure. *below*, longer exposure. **B**, Quantification of relative expression of each TRPA1 construct relative to WT, normalized to β-actin. Points represent normalized values from *n* = 3 independent experiments.

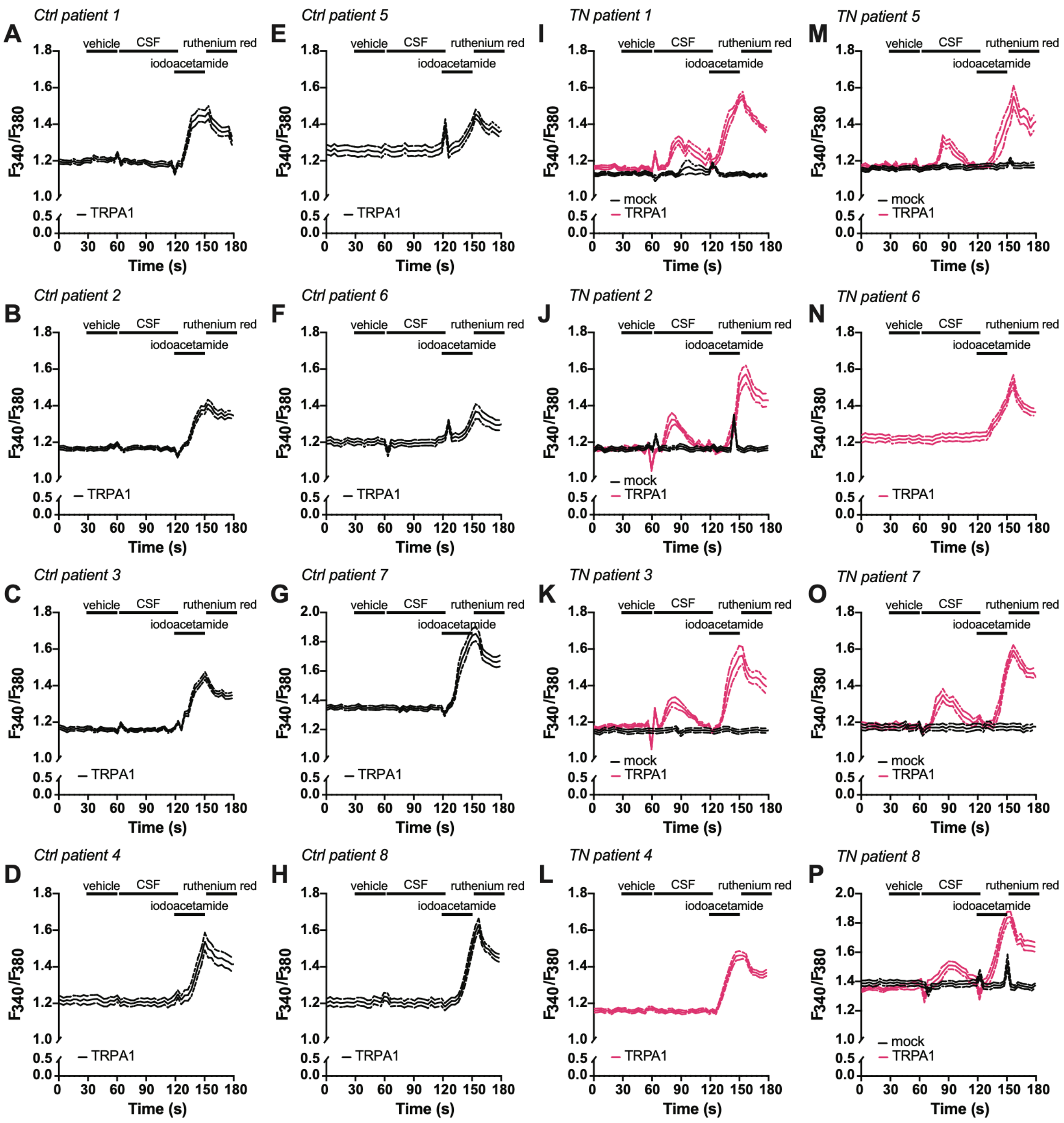

Figure S3. TRPA1 selectively responds to CSF from patients with trigeminal neuralgia. **A-P**, Calcium traces from HEK-293 cells transiently expressing WT TRPA1 in response to CSF from (*A-H*) control (Ctrl) and (*I-P*) trigeminal neuralgia (TN) patients. CSF was diluted into calcium imaging buffer 1:50 prior to each trial. As indicated by black bars, baseline signal was established for 30 s, after which cells were treated with vehicle. CSF was then applied for 60 s, after which cells were treated with 100 µM iodoacetamide. 50 µM of the non-selective TRP channel inhibitor ruthenium red was applied for 30 s at the end of every imaging trial. (*A-P*) Mean ± 95% CI depicted with dashed lines.

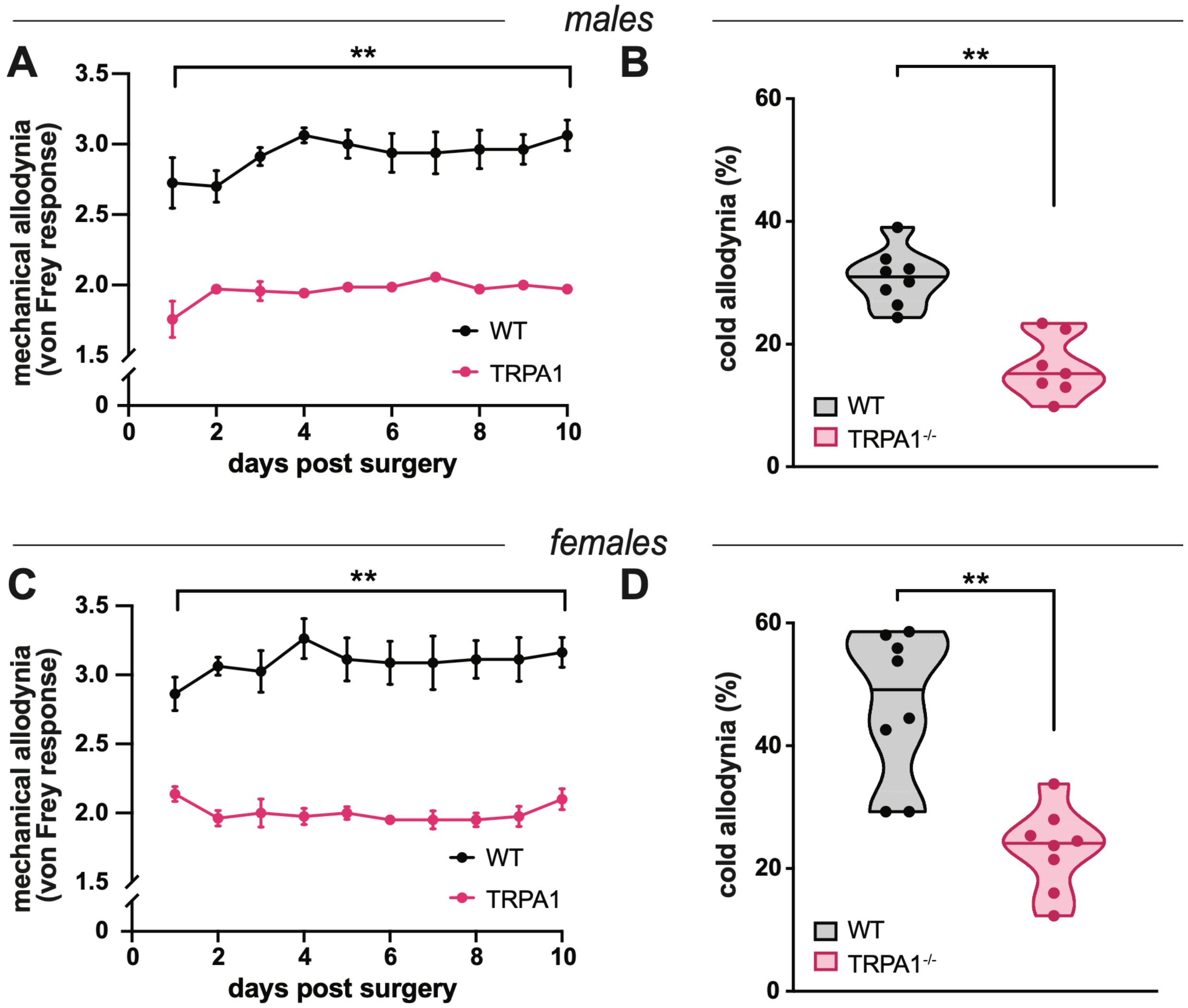

Figure S4. TRPA1 null males and females both exhibit less mechanical and thermal pain. **A-B**, (*A*) Scored mechanical allodynia and (*B*) timed cold allodynia from male WT and TRPA1$^{-/-}$ mice following constriction of the maxillary nerve. Points in (*A*) represent the mean ± SEM of n = 8 (WT) and 7 (TRPA1$^{-/-}$). Points in (*B*) represent individual mice. **B-C**, (*A*) Scored mechanical allodynia and (*B*) timed cold allodynia from female WT and TRPA1$^{-/-}$ mice following constriction of the maxillary nerve. Points in (*A*) represent the mean ± SEM of n = 8 (WT) and 8 (TRPA1$^{-/-}$). Points in (*B*) represent individual mice. (*A* and *C*) Mean ± 95% CI depicted with dashed lines. (*B* and *D*) Median and range depicted. ** = P < 0.01 by two-tailed unpaired Student's t-test.

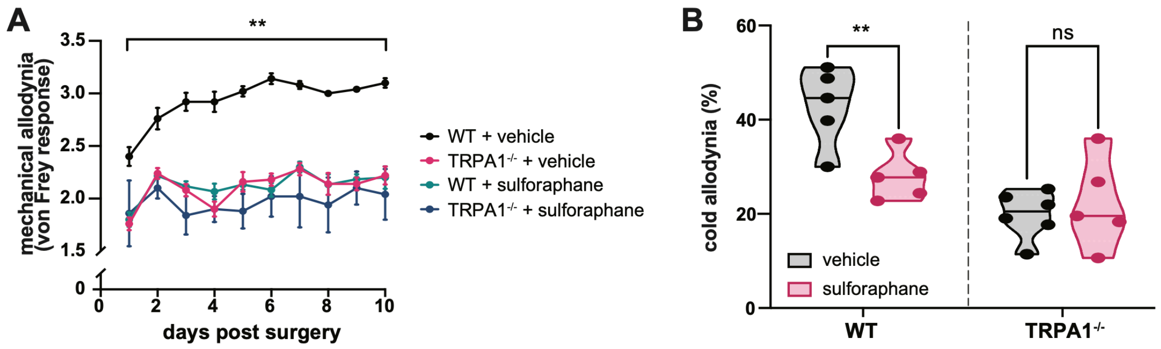

Figure S5. Sulforaphane does not comparatively lower mechanical or cold allodynia in TRPA1-/- mice.

A-B, (*A*) Scored mechanical allodynia and (*B*) timed cold allodynia from WT and TRPA1$^{-/-}$ mice that underwent constriction of the maxillary nerve. Mice were treated with either vehicle or sulforaphane (10 mg/kg, i.p.) daily for two days before surgery and again daily just after behavior testing. Points in (*A*) represent the mean ± SEM of *n* = 5 (WT, vehicle), 5 (TRPA1$^{-/-}$, vehicle), 6 (WT, sulforaphane), and 5 (TRPA1$^{-/-}$, sulforaphane). Points in (*B*) represent individual mice. (*B*) Median and range depicted. ** = *P* < 0.01 and ns = *P* > 0.05 by two-tailed unpaired Student's t-test.

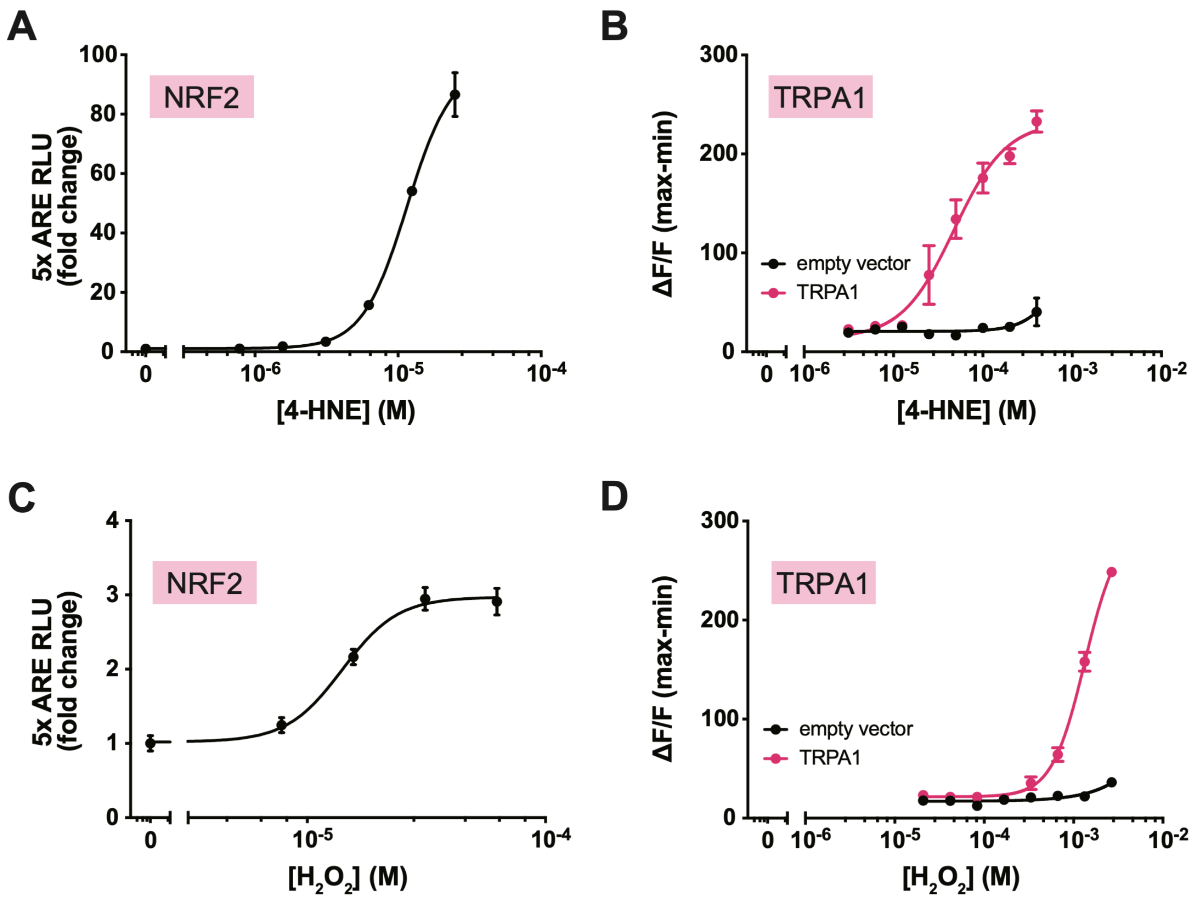

Figure S6. 4-HNE and H_2O_2 activate NRF2 at lower concentrations than TRPA1.

A-D, (*A* and *C*) NRF2-dependent luciferase activity and (*B* and *D*) concentration–Ca2+ response curves after varying concentrations of (*A*-*B*) 4-HNE and (*C*-*D*) H_2O_2. Cells in (*A* and *C*) were treated for 6 hours to allow for transcription and translation of luciferase. HEK-293 cells in (*B* and *D*) were transfected with either WT TRPA1 cDNA or the empty vector. Data are a representative experiment of 2-4 independent experiments performed in triplicate, depicted as mean ± SEM.

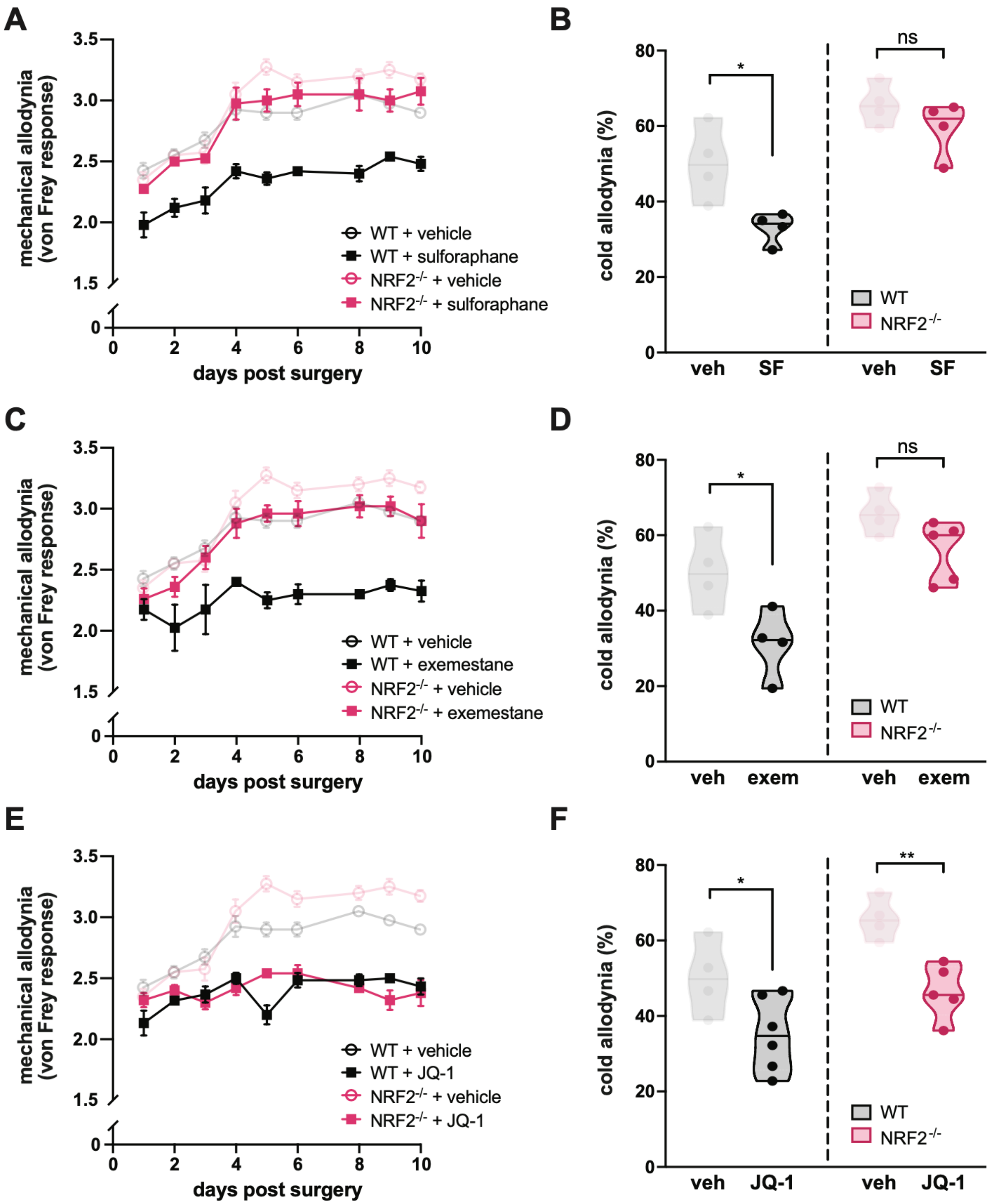

Figure S7. WT and NRF2-/- mice treated with either sulforaphane, exemestane, or JQ-1. **A-F**, (*A*, *C*, and *E*) Scored mechanical allodynia and (*B*, *D*, and *F*) timed cold allodynia from WT and NRF2$^{-/-}$ mice that underwent constriction of the maxillary nerve or sham surgery. Mice that underwent constriction were treated with either vehicle, (*A*-*B*) sulforaphane (10 mg/kg, i.p.), (*C*-*D*)

exemestane (10 mg/kg, i.p.), or (*E*-*F*) JQ-1 (40 mg/kg, i.p.) daily for two days before surgery and again daily just after behavior testing. Mice treated with sulforaphane, exemestane, or JQ-1 were all compared to the same vehicle-treated mice of the matching genotype regardless of drug treatment (uniformly depicted with reduced opacity in each plot). Points in (*A*-*F*) representing vehicle mice are measurements from n = 4 (WT) or n = 4 ($NRF2^{-/-}$). Points in (*A*, *C*, and *E*) represent the mean ± SEM of vehicle-treated mice and: in (*A*), n = 5 (WT) and 4 ($NRF2^{-/-}$); in (*C*), n = 4 (WT) and 5 ($NRF2^{-/-}$); in (*E*), n = 6 (WT) and 5 ($NRF2^{-/-}$). Points in (*B*, *D*, and *F*) represent individual mice. Median and range depicted. ** = $P < 0.01$, * = $P < 0.05$, and ns = P > 0.05 by two-tailed unpaired Student's t-test.

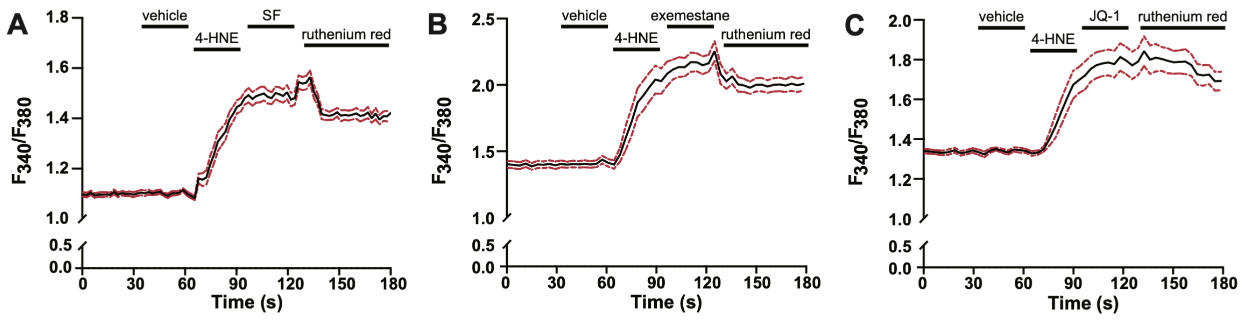

Figure S8. Neither sulforaphane, exemestane, nor JQ-1 inhibit TRPA1.

A-C, Calcium imaging of HEK-293 cells transiently expressing WT TRPA1. Cells were imaged for 30 s to establish a baseline, after which 4-HNE was applied for 30 s. As indicated by black bars, either (*A*) 10 µM sulforaphane (SF), (*B*) 1 µM exemestane or (*B*) 100 µM JQ-1 was then applied. 50 µM of the non-selective TRP channel inhibitor ruthenium red was applied for 30 s at the end of every imaging trial. Mean ± 95% CI depicted with dashed lines.

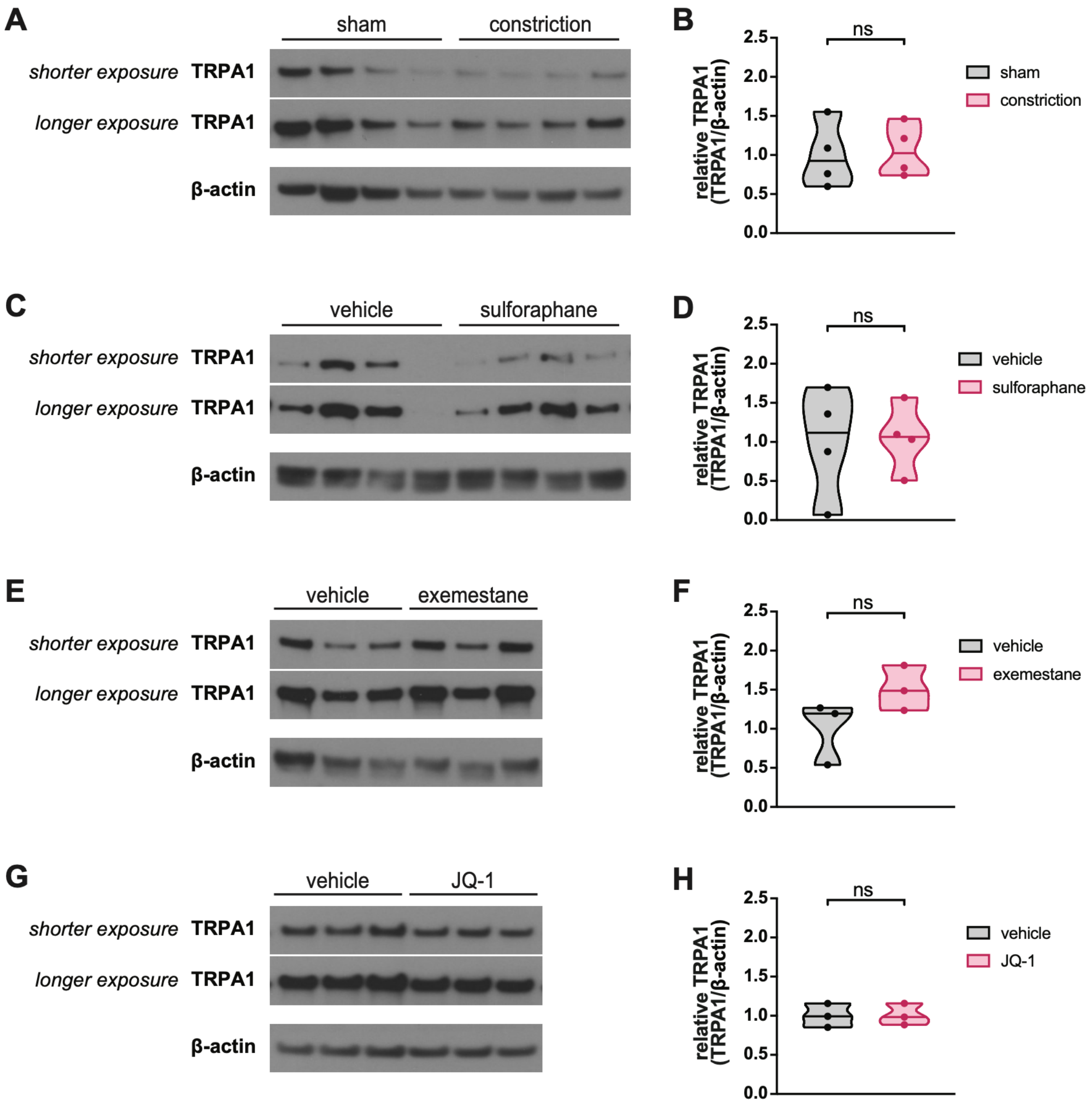

Figure S9. TRPA1 expression does not change with constriction, sulforaphane, exemestane, or JQ-1.

A-H, Immunoblot for TRPA1 from maxillary nerves of mice following constriction compared to (*A*) sham surgery or treatment with (*C*) sulforaphane, (*E*) exemestane, or (*G*) JQ-1. *above*, shorter exposure. *below*, longer exposure. Relative TRPA1 expression following constriction compared to (*B*) sham surgery or treatment with (*D*) sulforaphane, (*F*) exemestane, or (*H*) JQ-1, normalized to β-actin. Lanes and points represent individual mice. (*B*, *D*, *F*, and *H*) Mean depicted. ns = $P > 0.05$ by two-tailed unpaired Student's t-test.

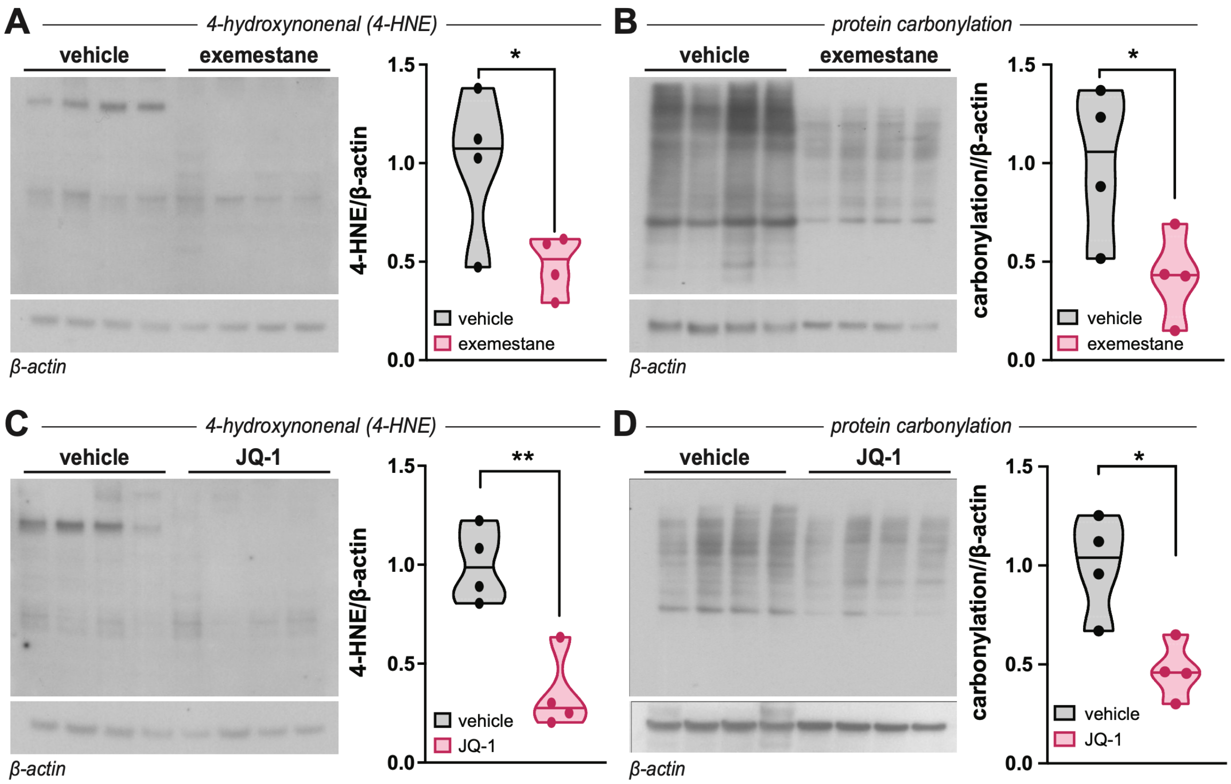

Figure S10. Exemestane and JQ-1 limit protein carbonylation and 4-HNE.

A-B, Immunoblots and quantification of (*A*) 4-HNE and (*B*) protein carbonylation from maxillary nerves of mice treated with either vehicle or exemestane after nerve constriction, normalized to β-actin. Lanes and points represent individual mice. **C-D**, Immunoblots and quantification of (*C*) 4-HNE and (*D*) protein carbonylation from maxillary nerves of mice treated with either vehicle or JQ-1 after nerve constriction, normalized to β-actin. Lanes and points represent individual mice. (*A-D*) Median and range depicted. * = $P < 0.05$, ** = $P < 0.01$, and ns = $P > 0.05$ by two-tailed unpaired Student's t-test

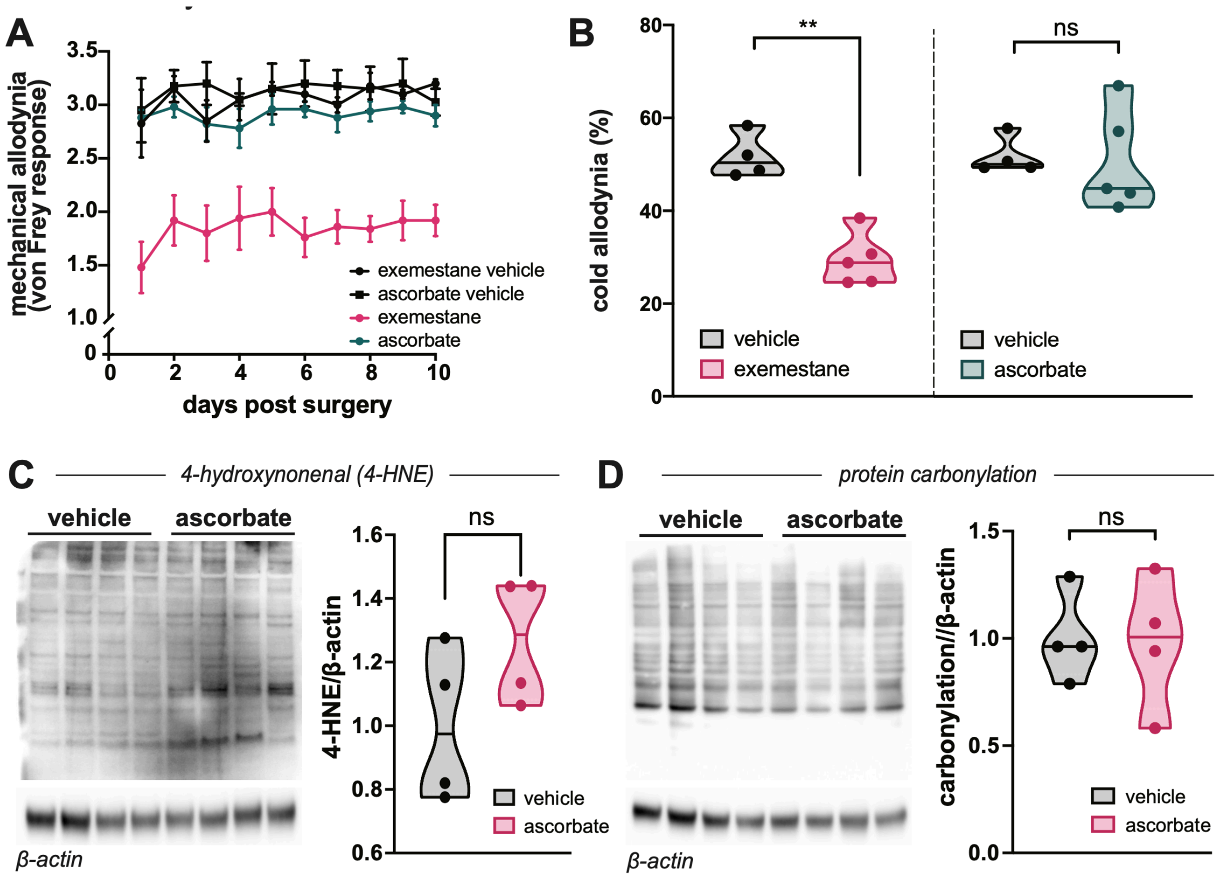

Figure S11. Non-specific antioxidant treatment does not alleviate mechanical or cold allodynia.

A-B, (*A*) Scored mechanical allodynia and (*B*) timed cold allodynia from mice that underwent constriction of the maxillary nerve. Mice were treated with either exemestane (10 mg/kg, i.p.), the exemestane vehicle, ascorbate (100 mg/kg, i.p.), or the ascorbate vehicle daily for two days before surgery and again daily just after behavior testing. Points in (*A*) represent the mean ± SEM of n = 5 (exemestane), 4 (exemestane vehicle), 5 (ascorbate), and 4 (ascorbate vehicle). Points in (*B*) represent individual mice. **C-D**, Immunoblots and quantification of (*C*) 4-HNE and (*D*) protein carbonylation from maxillary nerves of mice treated with either vehicle or ascorbate after nerve constriction, normalized to β-actin. Lanes and points represent individual mice. (*B-D*) Median and range depicted. ** = $P < 0.01$ and ns = $P > 0.05$ by two-tailed unpaired Student's t-test.

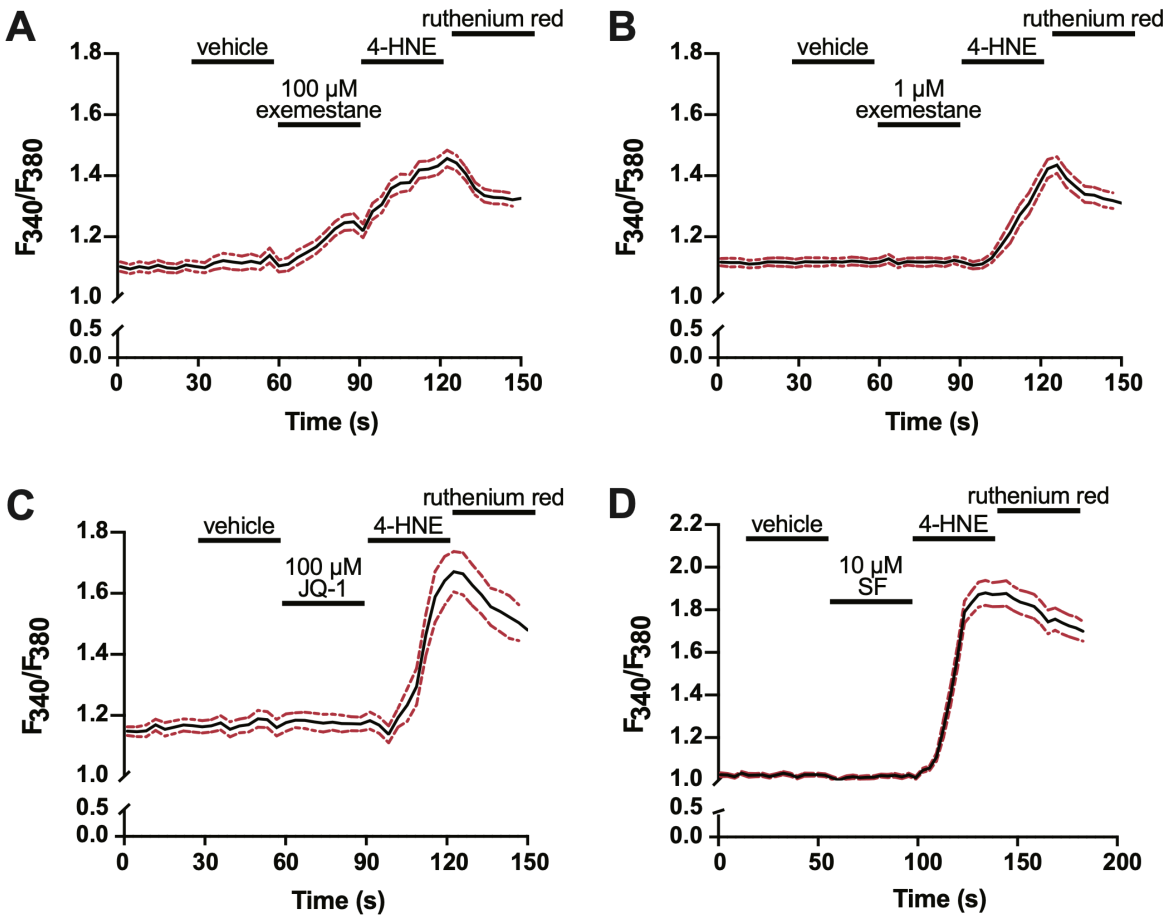

Figure S12. Therapeutic concentrations of exemestane, JQ-1, and sulforaphane do not activate TRPA1.

A-D, Calcium imaging of HEK-293 cells transiently expressing WT TRPA1. Cells were imaged for 30 s to establish a baseline, after which vehicle was applied for 30 s. As indicated by black bars, either (*A*) 100 µM exemestane, (*B*) 1 µM exemestane, (*C*) 100 µM JQ-1, or (*D*) 10 µM sulforaphane (SF) was then applied. 4-HNE was applied afterwards to identify TRPA1-expressing cells. 50 µM of the non-selective TRP channel inhibitor ruthenium red was applied for 30 s at the end of every imaging trial. (*A-D*) Mean ± 95% CI depicted with dashed lines.

Table S1. Compound Connectivity Scores for *Nfe2l2*-derived and *Keap1*-derived transcriptome signatures.

Table S2. Differential gene expression in primary human dermal fibroblasts treated with vehicle or JQ-1.

Table S3. Gene ontology analysis of JQ-1-dependent differential gene expression.
